# A Conserved Fibroblast-Myeloid Gene Signature in Digestive Cancers: Multi-Omics Integration Identifies *DCN*, *COL10A1*, *CTHRC1*, and *TREM2* as Candidate Microenvironmental Markers

**DOI:** 10.3390/ijms27073208

**Published:** 2026-04-01

**Authors:** Changyi Li, Yimu Yang, Wenxia Zhang, Haili Wang, Yingle Liu, Qi Zhang

**Affiliations:** 1 State Key Laboratory of Virology, College of Life Sciences, Wuhan University, Wuhan 430072, China; 2019302040013@whu.edu.cn (C.L.); 2018302040089@whu.edu.cn (Y.Y.); 2019202040047@whu.edu.cn (W.Z.); 2020202040044@whu.edu.cn (H.W.); 2 Frontier Science Center for Immunology and Metabolism, Wuhan University, Wuhan 430072, China

**Keywords:** digestive cancers, multi-omics analysis, fibroblasts, myeloid, *DCN*, *COL10A1*, *CTHRC1*, *TREM2*

## Abstract

Digestive cancers exhibit high heterogeneity and poor prognosis, yet whether their tumor microenvironments share conserved stromal–immune interactions remains unclear. Here, we performed an integrative multi-omics analysis across seven digestive cancer types and identified a conserved four-gene signature—*DCN*, *COL10A1*, *CTHRC1*, and *TREM2*—that is consistently enriched in matrix cancer-associated fibroblasts (mCAFs) and myeloid cells. Single-cell RNA sequencing revealed that *DCN*, *COL10A1*, and *CTHRC1* are predominantly expressed in mCAFs, while *TREM2* is enriched in myeloid cells and, to a lesser extent, in antigen-presenting CAFs(apCAFs). Cell–cell communication analysis consistently identified a fibroblast-to-myeloid signaling network centered on ECM-CD44 interactions across all examined cancer types, providing a candidate framework for intercellular crosstalk. Multi-omics profiling further characterized the genomic, epigenetic, and immune correlates of this signature. Collectively, these findings identify a conserved stromal–myeloid gene signature across digestive cancers and provide a candidate gene set for future diagnostic and therapeutic exploration.

## 1. Introduction

Digestive cancers, including cholangiocarcinoma, esophageal cancer, hepatocellular carcinoma, gastric cancer, pancreatic cancer, and colorectal cancer, among others, are a major cause of global cancer-related morbidity and mortality. They exhibit significant heterogeneity in their etiology, molecular subtypes, and clinical outcomes, which poses substantial challenges for developing universal diagnostic and therapeutic strategies, thereby placing a heavy burden on global healthcare systems. According to statistics from 2022, the global prevalence rates for colorectal, gastric, and liver cancers ranked third, fifth, and sixth, respectively, at 9.6%, 4.9%, and 4.3%. Furthermore, the prevalence rates for other digestive tract-related cancers such as esophageal cancer, pancreatic cancer, and gallbladder cancer were 2.6%, 2.6%, and 0.6%, respectively. Patients with digestive tract cancers account for nearly a quarter of the total cancer population, and deaths from digestive tract cancers constitute one-third of all cancer-related deaths [1]. However, due to the heterogeneity of cancer, there are currently no unified diagnostic and therapeutic methods for digestive tract cancers.

From a developmental biology perspective, the digestive tract originates from the endoderm during embryonic development. The formation of all endodermal organs relies on interactions between the endodermal epithelium and the surrounding mesodermal mesenchyme, and all are regulated by signaling pathways such as Wnt and Notch, indicating a degree of similarity [2].

Therefore, we sought to investigate the similarities among digestive cancers. To this end, we analyzed seven types of digestive cancers and identified four genes—*DCN*, *COL10A1*, *CTHRC1*, and *TREM2*. Using multiple computational methods, we found these genes are primarily active in specific cell types within the TME, particularly cancer-associated fibroblasts (CAFs) and immune cells. Our multi-omics analysis revealed that these genes participate in critical cancer processes including tissue remodeling and immune regulation. This four-gene signature provides new insights for developing diagnostic tools and targeted therapies for digestive cancers.

## 2. Results

### 2.1. A Conserved Co-Expression Module Emerges from Pan-Digestive Cancer Transcriptomes

**Figure 1:**
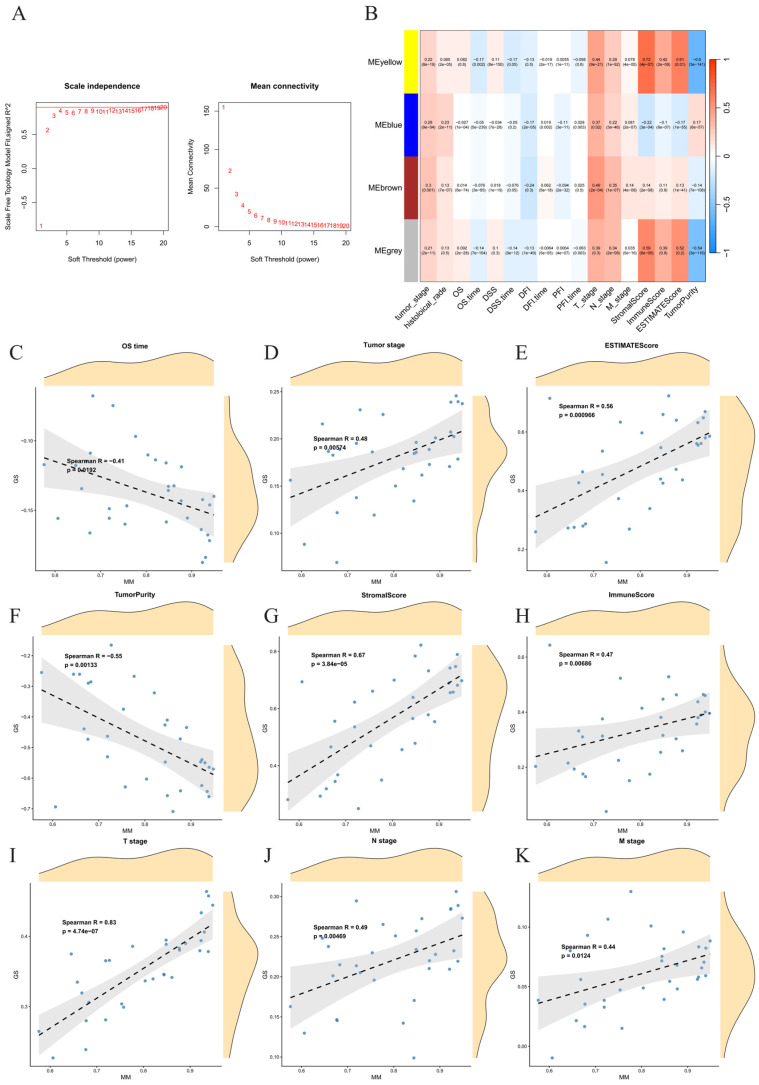
Identification of key modules by WGCNA. (**A**) Selection of a soft-thresholding power of 12 based on the scale-free topology criterion with a threshold of 0.9. (**B**) Heatmap showing the correlations between module eigengenes and clinical traits. The yellow module demonstrated the highest correlations with clinical traits. (**C**–**K**) Scatter plots of Gene Significance (GS) versus Module Membership (MM) in the yellow module for various clinical traits, showing strong correlations for all traits.

Differential expression analysis across seven digestive cancers identified 540 consistently dysregulated protein-coding genes (Supplementary Table S1). Weighted Gene Co-expression Network Analysis (WGCNA) was performed on these 540 genes. A soft power threshold of 12 was selected to ensure a scale-free topology (Figure 1A), resulting in four distinct modules. The grey module, representing non-co-expressed genes, was excluded from further analysis. Among the remaining three modules, the yellow module demonstrated the highest correlation with clinical traits (Figure 1B) and was selected for subsequent studies, encompassing 32 genes. Significant correlations between Gene Significance (GS) and Module Membership (MM) were observed for all clinical indices within the yellow module (Figure 1C–K), indicating a pronounced association with clinical features.

### 2.2. The Conserved Module Is Functionally Linked to ECM Remodeling, EMT, and Poor Prognosis

**Figure 2:**
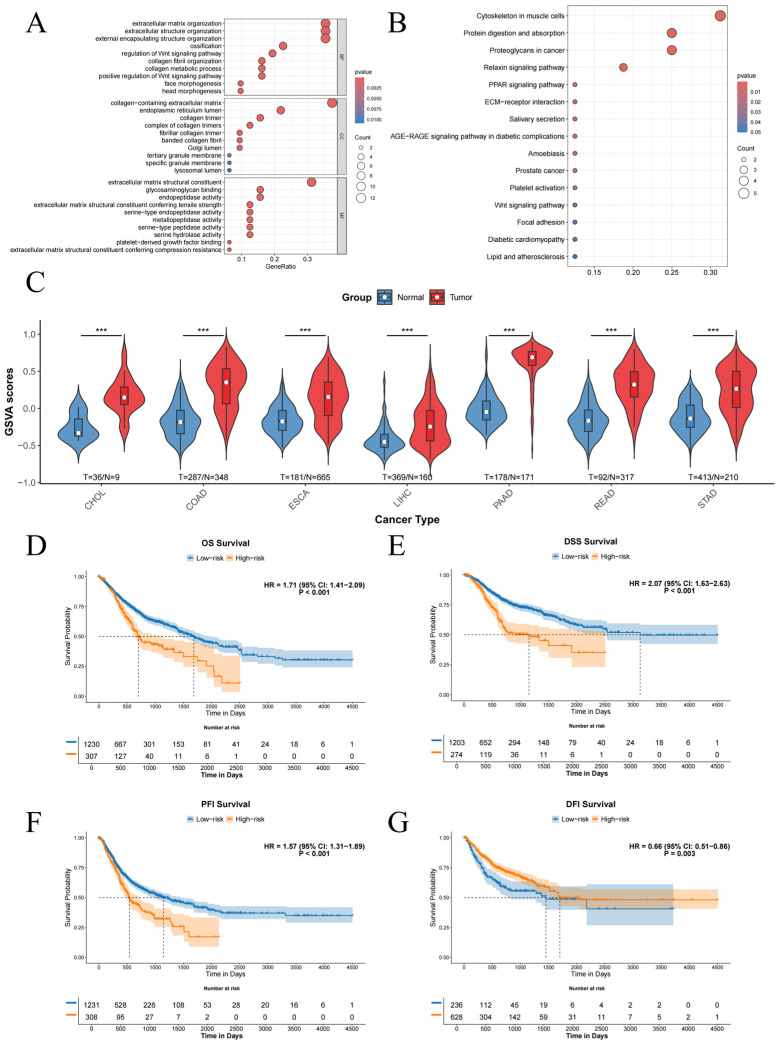
Functional analysis of the key WGCNA module gene set. (**A**) Results of the Gene Ontology (GO) enrichment analysis. (**B**) Results of the Kyoto Encyclopedia of Genes and Genomes (KEGG) enrichment analysis. (**C**) Violin plot showing the difference in GSVA scores between tumor and normal tissue samples(*p* < 0.001 is marked as ***). (**D**–**G**) Kaplan–Meier survival curves for OS, DSS, PFI, and DFI comparing high and low GSVA score groups, stratified by the optimal cutoff value and applied permutation test.

GO and KEGG enrichment analyses of the 32 genes revealed significant enrichment in pathways related to the extracellular matrix(ECM) (Figure 2A,B). KEGG analysis further indicated enrichment in cancer-associated pathways, such as the Wnt signaling pathway. GSVA using this 32-gene set showed significantly higher scores in tumor tissues compared to normal tissues across all seven cancers (Figure 2C). Survival analysis demonstrated a significant difference in OS between high-risk and low-risk groups defined by the GSVA score (Figure 2D–G). Furthermore, individual survival analysis for each gene across various survival states and cancer types revealed that most genes were associated with patient prognosis (Supplementary Figure S1).

**Figure 3:**
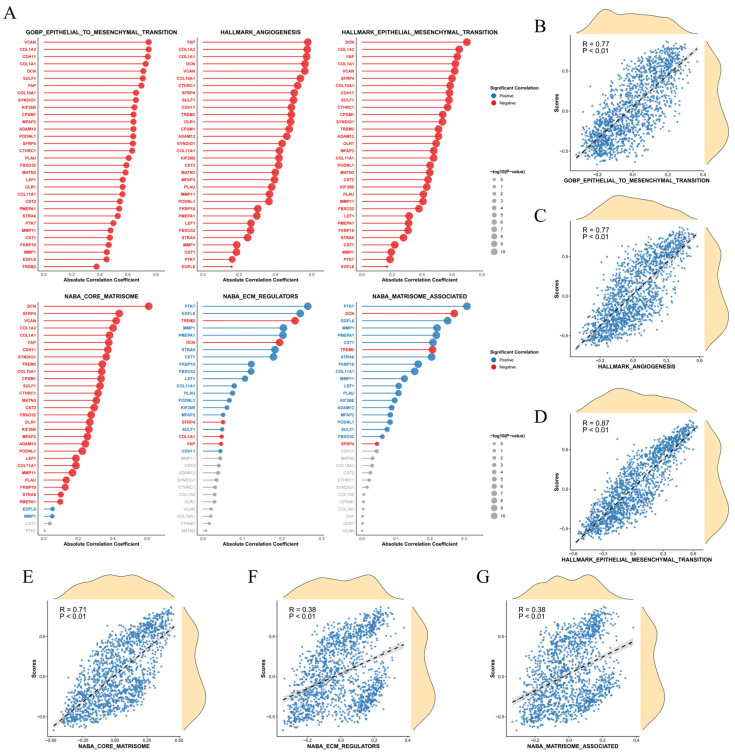
Associations between the custom gene set and public gene sets. (**A**) Lollipop plot showing correlations between the key WGCNA module and gene sets related to epithelial–mesenchymal transition (EMT), angiogenesis, etc. Positive correlations are marked in red and negative correlations in blue. The length of a line represents the absolute value of the correlation coefficient, and the size of a circle represents the significance level (*p*-value). (**B**–**G**) Scatter plots showing the correlations between the GSVA score of the custom gene set and the GSVA scores of public gene sets.

Given the strong ECM association and established roles of EMT(Epithelial-mesenchymal transition) and angiogenesis in cancer, we investigated the relationship between our gene set and relevant gene sets from MSigDB. Our custom gene set showed significant positive correlations with established gene sets for EMT and angiogenesis (e.g., GOBP_EPITHELIAL_TO_MESENCHYMAL_TRANSITION, HALLMARK_ANGIOGENESIS, HALLMARK_EPITHELIAL_MESENCHYMAL_TRANSITION) (Figure 3A). The GSVA scores of these gene sets were also significantly correlated with our custom gene set score (Figure 3B–G), suggesting that our identified genes are potentially involved in EMT and angiogenic processes during tumorigenesis.

### 2.3. A Cross-Method Consensus Identifies Four Hub Genes

**Figure 4:**
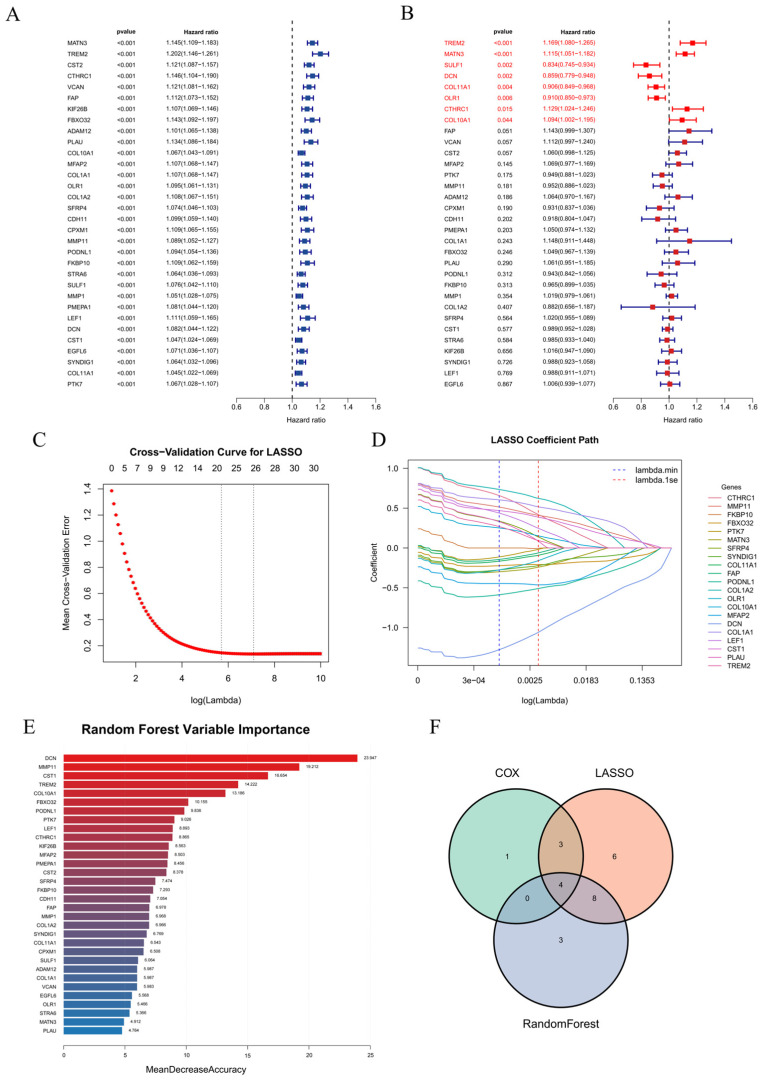
Screening of hub genes. (**A**) Results of the univariate Cox regression analysis. (**B**) Results of the multivariate Cox regression analysis (genes with *p* value < 0.05 are marked in red). (**C**) Cross-validation plot for the LASSO regression. (**D**) LASSO coefficient profile plot. (**E**) Bar plot of variable importance from the random forest algorithm. (**F**) Venn diagram showing the intersection of the three methods, identifying four genes (*DCN*, *COL10A1*, *CTHRC1*, *TREM2*).

To identify the critical genes among the 32 genes, we employed univariate Cox regression, multivariate Cox regression, LASSO regression, and random forest analysis. Univariate Cox analysis showed that all 32 genes were significantly associated with overall survival (OS) (Figure 4A). Multivariate Cox analysis identified eight genes with *p* values < 0.05 (Figure 4B). LASSO regression selected 21 genes with non-zero coefficients at lambda.1se (Figure 4C,D). The random forest algorithm ranked the top 15 genes by variable importance (Figure 4E). The intersection of these four analyses yielded four hub genes: *DCN*, *COL10A1*, *CTHRC1*, and *TREM2* (Figure 4F). We stratified the samples into high-risk and low-risk groups based on the respective expression levels of the four genes. Survival analysis showed that OS time differed significantly between the high-risk and low-risk groups (Supplementary Figure S2).

### 2.4. Single-Cell Resolution Uncovers Distinct Cellular Origins in mCAFs and Myeloid Compartments

**Figure 5:**
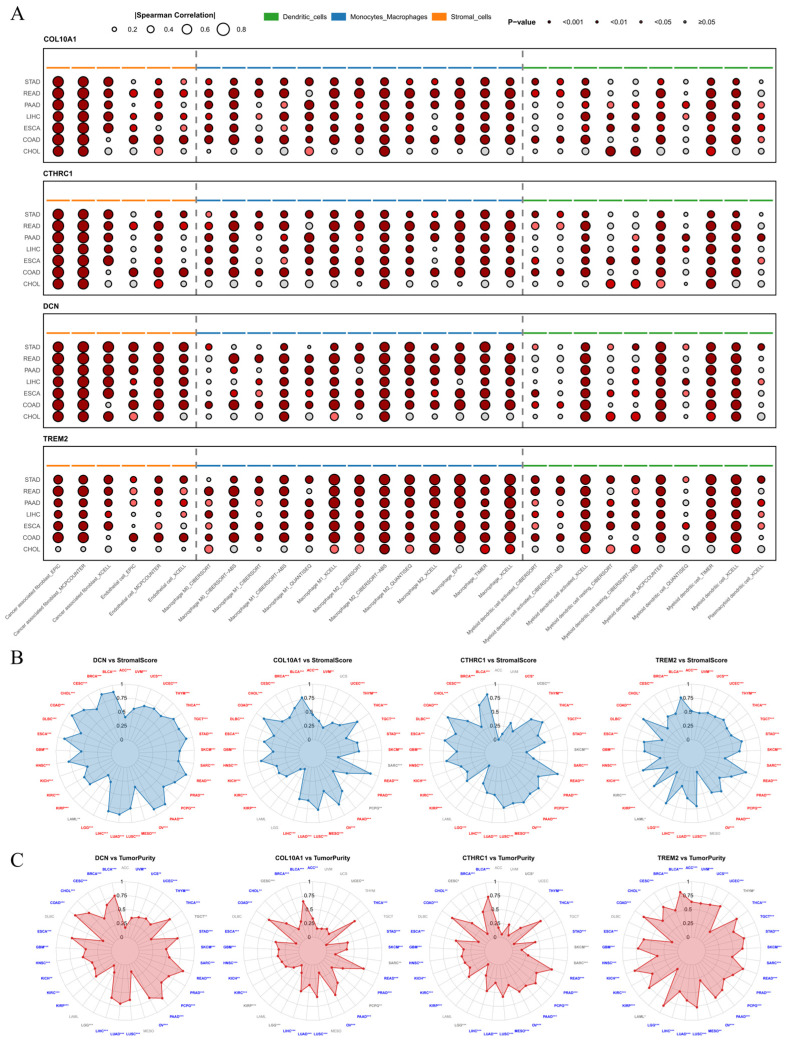
Immune infiltration analysis. (**A**) Correlation of *DCN*, *COL10A1*, *CTHRC1*, and *TREM2* expression with stromal and myeloid immune cell infiltration levels across digestive cancers. The x-axis shows different immune deconvolution methods (TIMER, EPIC, MCP-COUNTER, XCELL, CIBERSORT, CIBERSORT-ABS, QUANTISEQ), and the y-axis shows cancer types. The size of a dot represents the absolute value of the correlation coefficient, and the color represents the significance level (*p*-value), with darker colors indicating more significant *p*-values and grey indicating non-significance. (**B**,**C**) Radar charts showing the correlations of *DCN*, *COL10A1*, *CTHRC1*, and *TREM2* with the ESTIMATE stromal score and tumor purity across various cancers. *p* < 0.05 is labeled as *, *p* < 0.01 as **, and *p* < 0.001 as ***. Cancer types with a significant positive correlation (r > 0.3) are marked in red, and those with a significant negative correlation (r < −0.3) are marked in blue.

Immune infiltration analysis indicated that the expression of *DCN*, *COL10A1*, *CTHRC1*, and *TREM2* was significantly correlated with stromal and myeloid cell scores, particularly those of CAFs and macrophages (Figure 5A). Furthermore, in other cancer types, the expression of these four genes was significantly associated with the ESTIMATE StromalScore and TumorPurity (Figure 5B,C), suggesting their potential as broad-spectrum cancer biomarkers.

**Figure 6:**
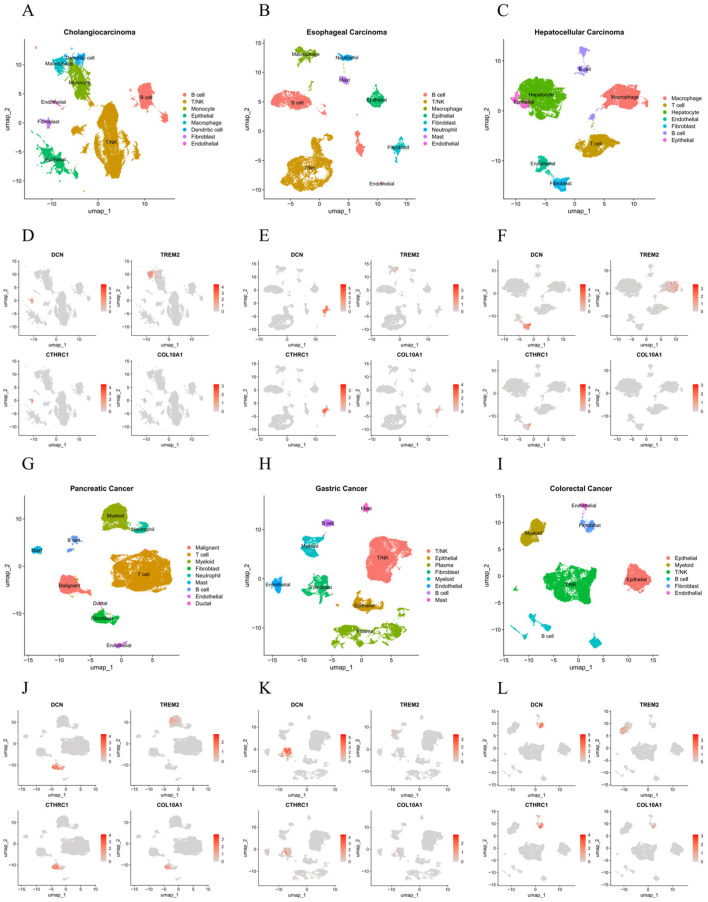
Single-cell RNA sequencing analysis: cell clustering and gene localization. (**A**–**C**,**G**–**I**) UMAP plots showing cell clustering in cholangiocarcinoma, esophageal carcinoma, hepatocellular carcinoma, pancreatic cancer, gastric cancer, and colorectal cancer. (**D**–**F**,**J**–**L**) Expression patterns of *DCN*, *COL10A1*, *CTHRC1*, and *TREM2* in cholangiocarcinoma, esophageal carcinoma, hepatocellular carcinoma, pancreatic cancer, gastric cancer, and colorectal cancer.

**Figure 7:**
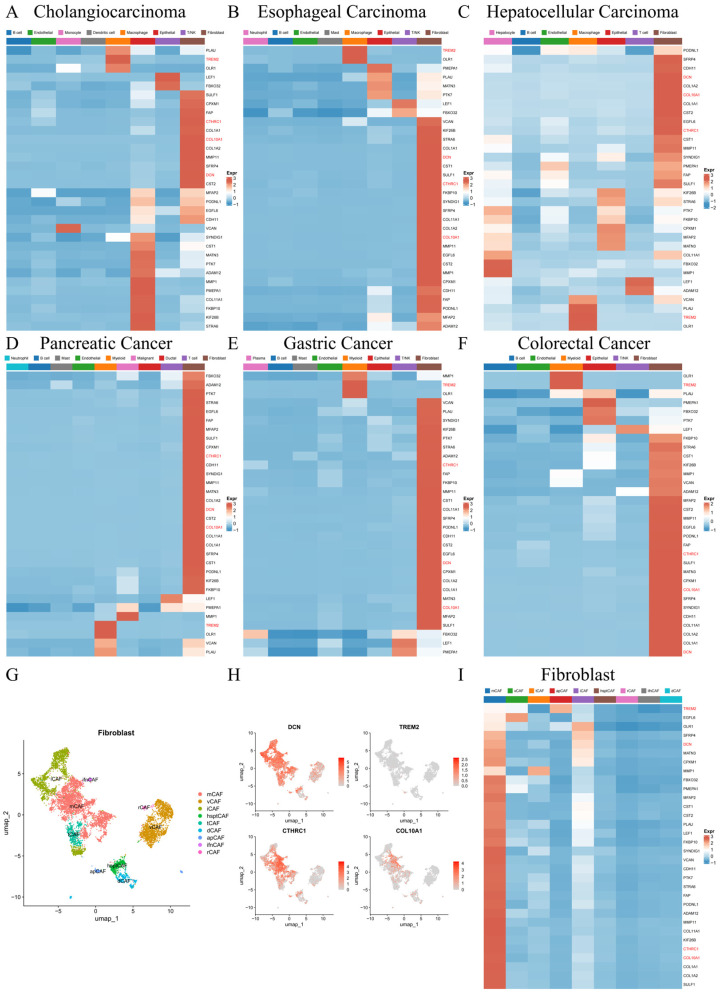
Gene expression heatmaps and fibroblast subclustering. (**A**–**F**) Heatmaps showing the expression of 32 genes in cholangiocarcinoma, esophageal carcinoma, hepatocellular carcinoma, pancreatic cancer, gastric cancer, and colorectal cancer, with expression levels increasing from blue to red. (**G**) UMAP plot of clustered fibroblasts. (**H**) Expression of *DCN*, *COL10A1*, *CTHRC1*, and *TREM2* in fibroblasts. (**I**) Heatmap showing the expression of the 32 genes in fibroblasts (*DCN*, *COL10A1*, *CTHRC1*, and *TREM2* are marked in red).

scRNA-seq analysis across digestive cancers revealed that *DCN*, *COL10A1*, and *CTHRC1* were predominantly expressed in fibroblasts, while *TREM2* was primarily expressed in myeloid cells, especially macrophages (Figure 6 and Figure 7A–F). The cellular composition was consistent across samples from each cancer type (Supplementary Figure S3). Further subclustering of fibroblasts showed that *DCN*, *COL10A1*, and *CTHRC1* were highly enriched in mCAFs (Figure 7G–I), which are known to maintain and remodel the ECM and promote EMT. *TREM2* was also found to be expressed at lower levels in apCAFs, implying a potential role in antigen presentation.

### 2.5. Multi-Omics Profiling Elucidates Genomic, Epigenetic, and Post-Transcriptional Regulation

**Figure 8:**
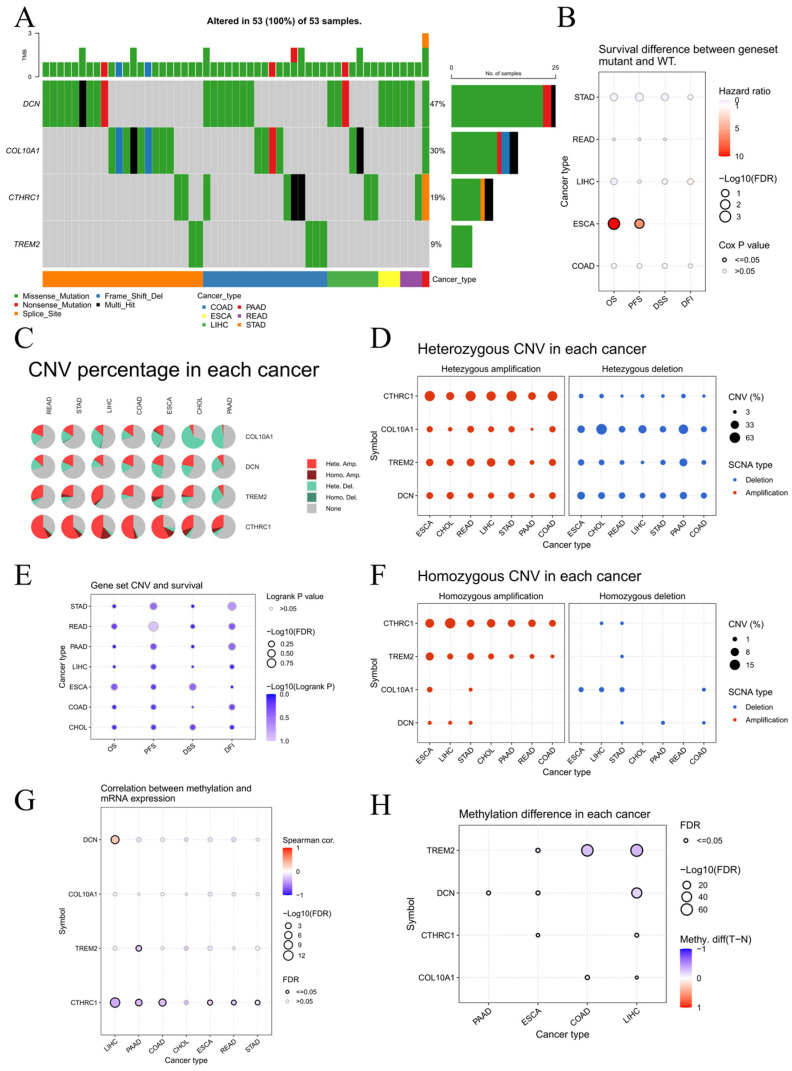
Genetic and epigenetic alterations of the hub genes. (**A**) Overview of SNV alterations in *DCN*, *COL10A1*, *CTHRC1*, and *TREM2* across digestive cancers. (**B**) Bubble chart comparing survival time between the mutation group (at least one gene mutated) and the normal group (no mutations in any gene). Bubble color from blue to red represents the hazard ratio from low to high. Bubble size is positively correlated with the significance of the Cox *p*-value. A black outline indicates Cox *p*-value ≤ 0.05. (**C**) Overview of copy number variation (CNV) for *DCN*, *COL10A1*, *CTHRC1*, and *TREM2* in digestive cancers. Hete.Amp., Homo.Amp., Hete.Del., and Homo.Del. represent the percentages of samples with heterozygous amplification, homozygous amplification, heterozygous deletion, and homozygous deletion, respectively. (**D**) Proportion of heterozygous CNV for each gene across digestive cancers. (**E**) Survival analysis based on the CNV status of the four-gene set. (**F**) Proportion of homozygous CNV for each gene across digestive cancers. (**G**) Correlation between mRNA expression and methylation levels for each gene. (**H**) Differences in methylation levels between tumor and adjacent normal tissues.

Genetic alteration analysis showed that single-nucleotide variations(SNVs) in these genes were predominantly missense mutations, with *DCN* having a relatively high mutation rate (Figure 8A). However, these SNVs had no significant impact on survival, except in esophageal carcinoma (Figure 8B). In contrast, copy number variations (CNVs), particularly amplifications, were frequent (Figure 8C,D,F) and significantly influenced patient survival (Figure 8E). Methylation analysis revealed a significant negative correlation between *CTHRC1* mRNA expression and its promoter methylation level in multiple cancers (Figure 8G), suggesting potential epigenetic regulation. Differential methylation was also observed between tumor and normal tissues for all four genes (Figure 8H).

**Figure 9:**
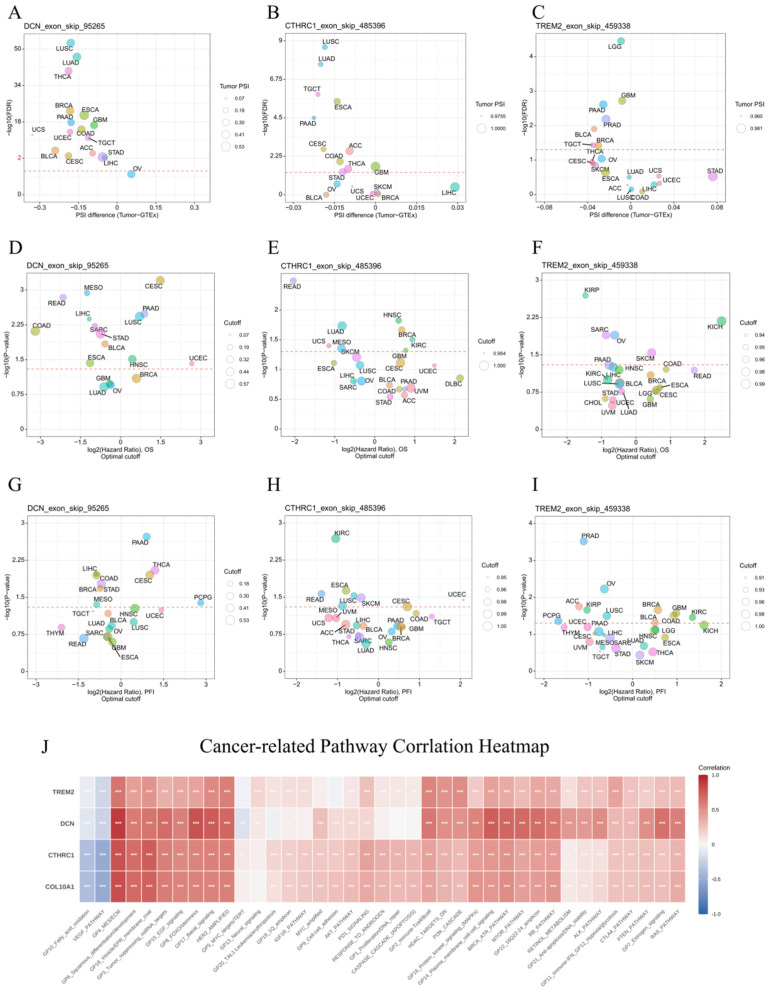
Alternative splicing and pathway correlation. (**A**–**C**) Differences in alternative splicing events for *DCN*, *CTHRC1*, and *TREM2* between TCGA tumor samples and GTEx normal samples. (**D**–**F**) Associations between alternative splicing events of *DCN*, *CTHRC1*, and *TREM2* and overall survival (OS). (**G**–**I**) Associations between alternative splicing events of *DCN*, *CTHRC1*, and *TREM2* and the progression-free interval (PFI). (AS data for *COL10A1* were unavailable.) (**J**) Heatmap showing the correlations between *DCN*, *CTHRC1*, *COL10A1*, *TREM2* and cancer-related pathways (*p* < 0.05 is labeled as *, *p* < 0.01 as **, and *p* < 0.001 as ***).

Alternative splicing (AS) analysis showed that *DCN* exhibited significantly different AS patterns between tumor and GTEx normal tissues, with generally fewer splicing events in tumors (Figure 9A). The frequency of *DCN* splicing events was associated with OS, acting as a risk factor in PAAD but a protective factor in READ, COAD, LIHC, STAD, and ESCA (Figure 9D). AS events for *CTHRC1* and *TREM2* were less pronounced and showed inconsistent associations with survival (Figure 9B,C,E,F). No significant associations were found between AS events and the progression-free interval (PFI) for any gene (Figure 9G–I).

Correlation analysis demonstrated that all four hub genes were significantly associated with multiple cancer-related pathways and gene sets, particularly the VEGF pathway (related to angiogenesis) and the GP4_MES/ECM gene set (Figure 9J). They also showed widespread significant correlations with immune-related molecules, including MHC molecules, chemokines, immune activators, immune suppressors, and immune checkpoint genes (Supplementary Figures S4 and S5). PPI network and enrichment analysis of their similar genes revealed that *DCN*, *COL10A1*, and *CTHRC1* were enriched in ECM-related pathways, while *TREM2* was enriched in immune receptor-related pathways (Supplementary Figure S6). In summary, our multi-omics approach confirmed that *DCN*, *COL10A1*, *CTHRC1*, and *TREM2* are linked to patient survival, likely through their involvement in EMT, angiogenesis, and antigen presentation, consistent with the scRNA-seq findings.

### 2.6. A Conserved ECM-CD44-Centered Fibroblast-Myeloid Communication Axis Across Digestive Cancers

**Figure 10:**
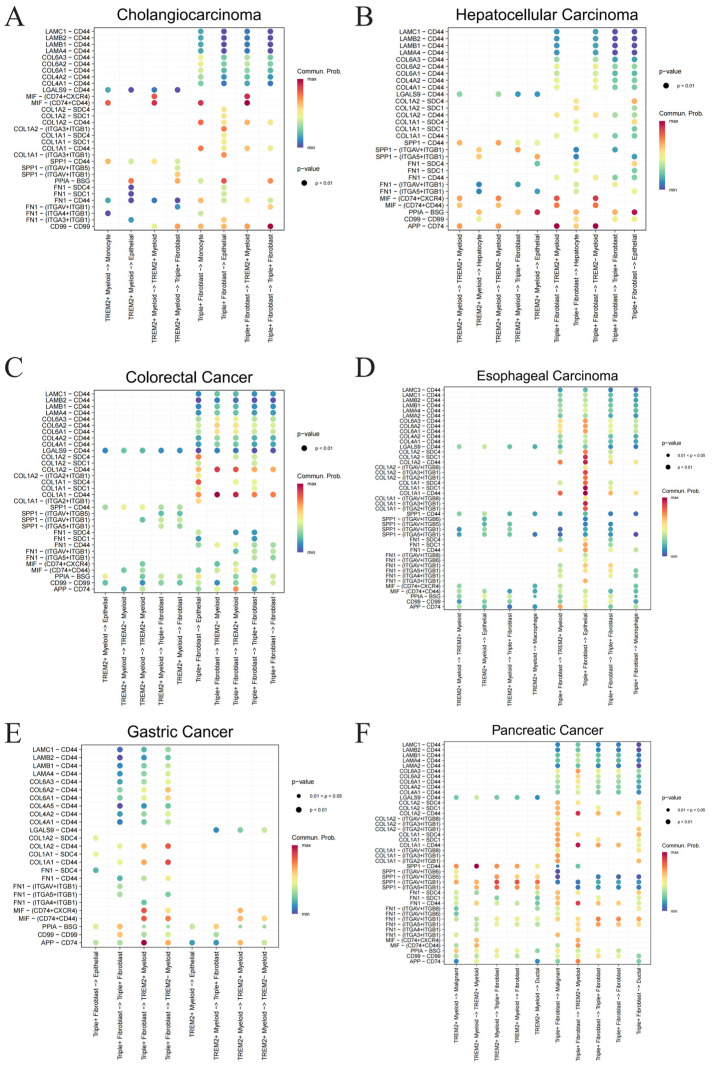
Cell–cell communication plots: (**A**) cholangiocarcinoma; (**B**) hepatocellular carcinoma; (**C**) colorectal cancer; (**D**) esophageal cancer; (**E**) gastric cancer; (**F**) pancreatic cancer.

The CellChat analysis revealed a highly conserved intercellular communication pattern across multiple digestive cancers, characterized by intensive bidirectional signaling between *CTHRC1*+ *DCN*+ *COL10A1*+ fibroblasts (Triple+ fibroblasts) and *TREM2*+ myeloid cells (Figure 10). Notably, Triple+ fibroblasts exhibited broad outgoing interactions not only toward *TREM2*+ myeloid cells but also toward epithelial/malignant cells and other stromal populations, indicating their central role as a signaling hub within the tumor microenvironment. Across all cancer types examined, a consistent set of ligand-receptor pairs was enriched, predominantly involving extracellular matrix (ECM)-related molecules, including FN1, SPP1, and multiple collagens (e.g., COL1A1, COL1A2, COL6A family), which interact with integrin complexes and the cell surface receptor CD44. Among these, CD44 emerged as a key convergent receptor, mediating interactions with a wide range of ECM components (FN1-CD44, SPP1-CD44, COL-CD44, LAMA/LAMB/LAMC-CD44), suggesting a central role in transducing stromal signals. In addition, immune-regulatory signaling pathways, such as MIF-(CD74 + CD44/CXCR4) and APP-CD74, were consistently observed, particularly in communications involving *TREM2*+ myeloid cells, further highlighting an immunomodulatory component.

Mechanistically, these findings lead us to propose a hypothesis that Triple+ fibroblasts actively remodel the ECM through the secretion of collagen, fibronectin, and related matrix proteins, thereby establishing a ligand-rich microenvironment that may engage CD44 and integrin receptors on both tumor and immune cells. Within this framework, the ECM-CD44/integrin signaling axis could potentially promote tumor cell plasticity, including enhanced migration and epithelial–mesenchymal transition, while also contributing to the polarization and maintenance of immunosuppressive *TREM2*+ myeloid populations via pathways such as MIF-CD74. In turn, *TREM2*+ myeloid cells may further reinforce fibroblast activation and matrix remodeling, forming a putative positive feedback loop that sustains a pro-tumorigenic and immune-suppressive niche. Importantly, the consistent involvement of ECM components and CD44-centered signaling across all examined cancer types supports the notion that this fibroblast-myeloid communication axis may represent a shared and potentially targetable mechanism underlying digestive cancer progression, although experimental validation is required to confirm this hypothesis.

### 2.7. A Powerful Diagnostic Model and Potential Therapeutic Implications Derived from Four Genes

**Figure 11:**
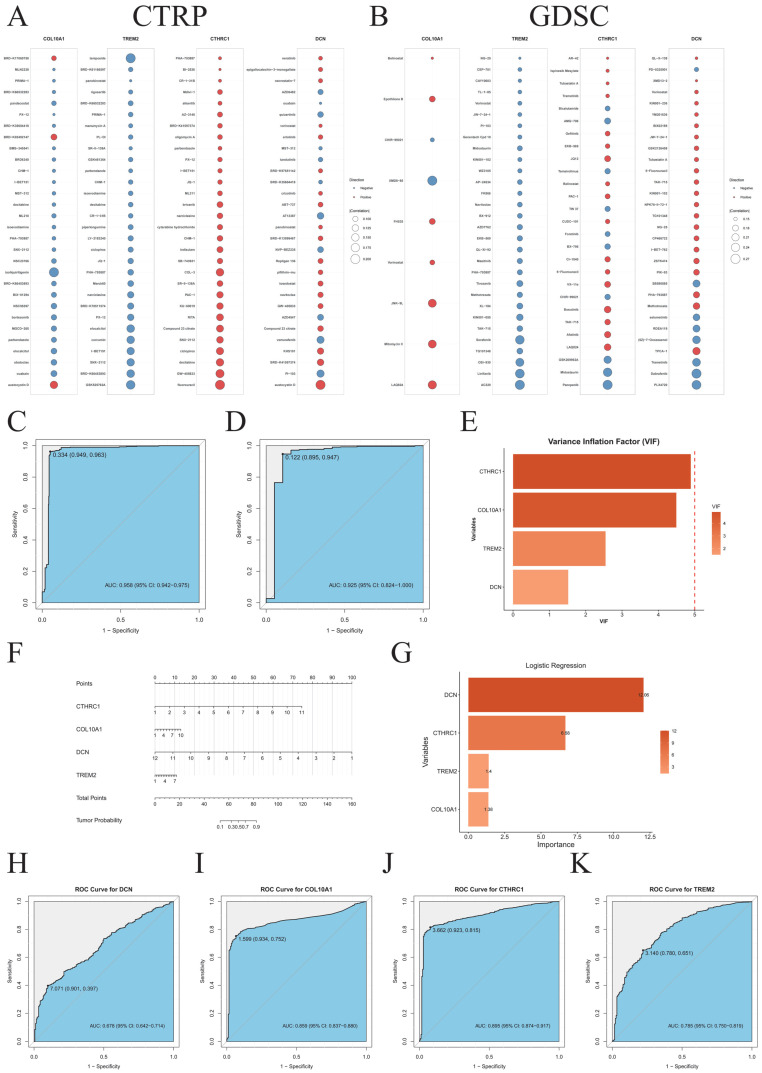
Drug sensitivity and diagnostic model performance. (**A**) Correlations between the expression of *DCN*, *CTHRC1*, *COL10A1*, and *TREM2* and drug IC50 values (based on the CTRP database). (**B**) Correlations between the expression of *DCN*, *CTHRC1*, *COL10A1*, and *TREM2* and drug IC50 values (based on the GDSC database). (**C**) ROC curve of the diagnostic model in the training set. (**D**) ROC curve of the diagnostic model in the validation set. (**E**) Bar plot of variance inflation factor analysis. (**F**) Nomogram of the diagnostic model. (**G**) Bar plot of variable importance in the diagnostic model. (**H**–**K**) ROC curves for the individual diagnostic performance of *DCN*, *COL10A1*, *CTHRC1*, and *TREM2*.

Analysis of drug sensitivity data from the CTRP [3] and GDSC [4] databases via GSCA revealed that the expression levels of *DCN*, *COL10A1*, *CTHRC1*, and *TREM2* were significantly correlated with the half-maximal inhibitory concentration(IC50) of multiple chemotherapeutic/targeted agents (Figure 11A,B), suggesting potential strategies for drug repurposing or combination therapy.

Furthermore, we constructed a logistic regression classification model based on TCGA data to distinguish normal samples from tumor samples, and we validated the model using datasets from the GEO database. The data from the TCGA and GEO databases were subjected to batch effect correction. A nomogram was developed to visualize the model (Figure 11F). The ROC curve showed that the AUC of the training set was 0.958 (95% CI: 0.942–0.975) (Figure 11C), while the AUC of the validation set was 0.925 (95% CI: 0.824–1.000) (Figure 11D). Variance inflation factor (VIF) analysis indicated that the VIF values of the four genes were all below 5 (Figure 11E), suggesting no significant multicollinearity. Variable importance analysis demonstrated that *DCN* and *CTHRC1* played relatively important roles in the model (Figure 11G). In addition, ROC curves were generated for each gene individually (Figure 11H–K). The results showed that, compared with single-gene diagnostic performance, the logistic regression model we constructed achieved superior performance.

## 3. Discussion

In this study, through various methods, we identified four genes that play important roles in digestive cancers. Using multi-omics technologies such as scRNA-seq, we determined that these genes are likely involved in processes such as ECM remodeling, EMT, vasculogenic mimicry, and immune responses.

Through immune infiltration analysis, we found that these four core genes are significantly associated with stromal cells and myeloid cells, particularly fibroblasts and macrophages. scRNA-seq revealed that *DCN*, *CTHRC1*, and *COL10A1* are primarily expressed in fibroblasts. Further subclustering of fibroblasts showed that they are mainly expressed in mCAFs. Additionally, genes similar to *DCN*, *CTHRC1*, and *COL10A1* were found to be significantly enriched in pathways related to extracellular matrix remodeling and maintenance. Research indicates that cancer-associated fibroblasts play multiple roles in the tumor microenvironment(TME) and are key players in shaping it, with functions including influencing tumorigenesis, inflammation, and remodeling of the ECM [5,6,7,8,9]. CAFs can be subdivided into mCAFs, iCAFs, and apCAFs, among others, with upregulated TGF-β signaling in mCAFs. This pathway is associated with KRAS signaling and EMT [10]. These findings suggest that *DCN*, *CTHRC1*, and *COL10A1* likely influence cancer growth and metastasis by participating in CAF-mediated ECM remodeling, EMT, and angiogenesis, which is consistent with the results of previous studies [11,12,13,14,15].

In contrast to *DCN*, *CTHRC1*, and *COL10A1*, *TREM2* is primarily expressed in myeloid cells such as macrophages, with minor expression in apCAFs. Although apCAFs are fibroblasts, they also play a role in tumor immunity, with significantly upregulated expression of MHC-II machinery-related genes [10], potentially involved in antigen presentation. This indicates that *TREM2* may play an important role in tumor immunity. Indeed, numerous studies have revealed the role of *TREM2* in tumor immunity, suggesting that it may sustain an immunosuppressive tumor microenvironment by promoting macrophage survival, proliferation, and metabolic adaptation, and loss of *TREM2* can inhibit tumor growth [16]. Another study on breast cancer lung metastasis also showed that *TREM2*+ macrophages have immunosuppressive functions [17]. This aligns with our findings that *TREM2* expression is significantly correlated with immunosuppressive genes such as *CSF1R*, *HAVCR2*, and *TGFB1* (Supplementary Figure S4C).

Building upon these cell-type-specific expression patterns, our cell–cell communication analysis further revealed a recurrent interaction pattern between fibroblast and myeloid populations across multiple digestive cancers. In particular, ECM-related ligand–receptor interactions, including those involving collagens, fibronectin (FN1), and SPP1, were consistently enriched and were predicted to occur with receptors such as CD44 and integrin complexes. In addition, signaling pathways such as MIF–CD74/CXCR4 were observed in communication involving myeloid cells. These results suggest that fibroblasts may contribute to shaping a ligand-rich microenvironment, which could influence both tumor cells and immune cells through ECM-related signaling. However, it should be noted that these findings are based on computational inference and therefore primarily provide a framework for potential intercellular interactions rather than direct evidence of functional signaling.

From a translational perspective, the logistic regression model we constructed demonstrated excellent diagnostic performance, indicating the great potential of this four-gene signature in distinguishing tumor from normal tissues. Additionally, the correlations between the expression of these genes and the IC50 values of various drugs may offer preliminary associations that could be explored in future studies regarding personalized therapy for patients with high expression of these genes.

Our study has several limitations. First, our findings are primarily based on bioinformatic analyses of public datasets and require further validation in independent clinical cohorts, as well as through in vitro and in vivo experiments. Second, the precise molecular mechanisms underlying the interactions of these genes within the tumor microenvironment remain unclear. For example, the specific role of mCAF in regulating EMT, and how *TREM2* + apCAFs interact with T cells, warrants further investigation. These unresolved questions should be addressed through functional studies, including gene editing techniques and co-culture experiments. In addition, although our multi-omics analysis demonstrated that *DCN*, *CTHRC1*, *COL10A1*, and *TREM2* play important roles in digestive cancers, several methodological limitations should be considered. Sensitivity analysis was not performed during gene selection using LASSO regression. Moreover, the use of arbitrary thresholds—such as selecting genes with *p* < 0.05 in Cox regression and the top 15 genes from the random forest—may have introduced bias, suggesting that the identified genes may not fully represent the most robust and core genes across digestive cancers. Furthermore, the validation cohort in the logistic regression model contained a relatively small number of normal samples, and data on alternative splicing for *COL10A1* were unavailable, which may also have affected the reliability and comprehensiveness of the results.

Overall, this study identified a recurrent stromal/myeloid-associated signature across digestive tumors, composed of genes with known relevance to the ECM and immune biology, and that these genes deserve follow-up validation. Through integrative multi-omics analyses, we showed that *DCN*, *COL10A1*, and *CTHRC1* are predominantly associated with fibroblast populations, particularly mCAFs, while TREM2 is mainly linked to myeloid cells. These genes are consistently correlated with ECM organization, EMT-related processes, and immune regulation and are further supported by inferred cell–cell communication patterns centered on ECM-related signaling. Collectively, these findings provide a systematic characterization of shared TME features across digestive cancers. However, given the primarily computational nature of this study, further experimental and clinical validation will be required to determine the functional roles and potential clinical relevance of this gene signature.

## 4. Materials and Methods

### 4.1. Screening of Differentially Expressed Genes (DEGs)

Transcriptomic data for seven cancer types—CHOL, COAD, ESCA, LIHC, PAAD, READ, and STAD—were obtained from the UCSC Xena database (dataset ID: TcgaTargetGtex_gene_expected_count). Differential expression analysis was performed for each cancer type using the ‘edgeR’ package (version: 4.6.3) in Rstudio (R version: 4.5.1) [18]. Genes with an absolute log2 fold change (|logFC|) > 1 and an adjusted *p*-value < 0.01 were considered significantly differentially expressed. The common DEGs across all seven cancer types were identified by taking the intersection of their respective DEG lists. Non-coding genes were subsequently removed, resulting in a final set of 540 conserved protein-coding DEGs for subsequent analysis.

### 4.2. ESTIMATE Scoring and WGCNA

The ‘ESTIMATE’ package in R was employed to calculate immune infiltration scores for each sample, generating the stromal score, Immune score, ESTIMATE score, and tumor purity [19]. WGCNA analysis was then conducted on the 540 DEGs using the ‘WGCNA’ package (version: 1.73) [20]. The scale-free topology criterion was set to 0.9, with a minimum module size of 25 genes and a merge cut height of 0.2. Modules exhibiting high correlations with clinical survival time, tumor stage, and ESTIMATE scores were identified. Ultimately, the yellow module, which demonstrated the most significant associations, was selected, and 32 genes from this module were extracted for further investigation.

### 4.3. Gene Set Enrichment and Custom GSVA Analysis

Gene Ontology (GO) and Kyoto Encyclopedia of Genes and Genomes (KEGG) pathway enrichment analyses were performed on the 32 selected module genes [21]. Gene Set Variation Analysis (GSVA) was utilized to evaluate the enrichment scores of the module gene set across samples. Differences in GSVA scores between tumor and normal tissues were assessed, survival analysis was conducted between high-score and low-score patient groups, and permutation tests were performed. Furthermore, gene sets related to EMT and endothelial–mesenchymal transition (EndMT) were downloaded from MSigDB for custom GSVA [22]. The correlations between the custom gene set scores, known gene set scores, and gene expression levels were systematically examined.

### 4.4. Identification of Core Genes

The 32 candidate genes were subjected to a rigorous multi-step screening process using Cox regression, LASSO regression, and random forest algorithms. In the univariate Cox regression analysis, genes with a *p*-value < 0.05 for overall survival (OS) were selected. These were subsequently included in a multivariate Cox regression to identify independent prognostic factors. For LASSO regression, the lambda.1se value (0.00215295) was applied, and genes with non-zero coefficients were retained. The random forest algorithm was used to rank genes based on variable importance, and the top 15 genes were selected. The intersection of the results from these three methods yielded four core genes: *DCN*, *COL10A1*, *CTHRC1*, and *TREM2*.

### 4.5. Immune Infiltration Analysis

The TIMER 2.0 web server was used to comprehensively analyze the correlations between the expression levels of the four core genes and immune cell infiltration levels in TCGA samples. This analysis incorporated multiple deconvolution algorithms, including TIMER [23], CIBERSORT [24], Quantiseq [25], xCell [26], MCP-counter [27], and EPIC [28].

### 4.6. Single-Cell RNA Sequencing (scRNA-Seq) Analysis and Cell–Cell Communication

scRNA-seq datasets for cholangiocarcinoma, esophageal carcinoma, hepatocellular carcinoma, pancreatic cancer, gastric cancer, and colorectal cancer were obtained from the GEO database; the detailed information is shown in Table 1. A standardized analytical pipeline was implemented, which included quality control (removing cells with >10% mitochondrial gene content or >1% hemoglobin gene content), doublet detection and removal using ‘DoubletFinder’ package (version: 2.0.6) [29], and regression of cell cycle effects. Data were then normalized, scaled, and subjected to principal component analysis (PCA). The top principal components capturing >90% of the cumulative variance were selected for non-linear dimensionality reduction (UMAP) and clustering. The ‘harmony’ package (version: 1.2.4) was applied to correct for batch effects [30]. Cell types were annotated based on canonical marker genes described in the literature [31,32,33,34,35,36]. To further investigate the expression of core genes within the tumor stroma, fibroblast populations were subsetted [10] and the entire analysis workflow (normalization, clustering, UMAP) was repeated on this specific cell type. Cell communication analysis was completed using the ‘CellChat’ package (version: 2.2.0) [37].

### 4.7. Mutation and Methylation Analysis

Genetic alteration and DNA methylation data for the core genes were retrieved from the GSCA database [38]. The mutation analysis encompassed both SNVs and copy CNVs, reporting the mutation frequency in patient samples and its impact on survival. Methylation analysis assessed the correlation between promoter methylation levels and mRNA expression, as well as differences in methylation between tumor and normal tissues.

### 4.8. Alternative Splicing Analysis

Data on alternative splicing events for *DCN*, *CTHRC1*, and *TREM2* were sourced from the OncoSplicing database (pancancer module) [39]; data for the *COL10A1* gene were unavailable. The analysis focused on identifying significant differences in splicing patterns between tumor and normal tissues and evaluating the prognostic impact of specific splicing events on patient survival.

### 4.9. Correlation with Cancer-Associated Pathways and Immune Factors

Data for cancer-related signaling pathway activities were acquired from UCSC Xena. Correlation analyses were performed to assess the relationships between the expression of the four core genes and the enrichment scores of these pathways. Additionally, the correlations between core gene expression and the expression levels of MHC molecules, chemokines, chemokine receptors, immune activators, immune suppressors, and immune checkpoint genes were systematically calculated.

### 4.10. Protein–Protein Interaction (PPI) Network Construction

The top 50 genes correlated with each core gene (*DCN*, *COL10A1*, *CTHRC1*, *TREM2*) were identified using GEPIA2 [40]. Each of these gene sets was submitted to the STRING database to construct a PPI network. The resulting interaction data were exported and visualized using R.

### 4.11. Drug Sensitivity Analysis and Diagnostic Model Construction

Data linking the mRNA expression of the core genes to drug IC50 values were obtained from the GSCA database Drug module [38], and correlation plots were generated in Rstudio.

We constructed a logistic regression model using data from the TCGA database. The TCGA data were obtained from UCSC Xena (dataset ID: tcga_RSEM_gene_tpm), and the model was further validated using the GEO dataset GSE39582. In the training dataset, there were 1562 tumor samples and 163 normal samples. Due to the substantial imbalance between the two classes, we applied both undersampling and oversampling techniques to achieve class balance and avoid model bias. After resampling, the final training set included 431 tumor samples and 431 normal samples. For external validation, the dataset comprised 566 tumor samples and 19 normal samples.

## Figures and Tables

**Table 1:** sc-RNAseq information.

Cancer Type	GEO Project Name	Sample ID	Cell Count
Cholangiocarcinoma	GSE201425	GSM6063506, GSM6063508, GSM6063510, GSM6063512, GSM6063514, GSM6063516, GSM6063518, GSM6063520, GSM6063522	36,458
Esophageal carcinoma	GSE196756	GSM5900215, GSM5900217, GSM5900219	12,359
Hepatocellular carcinoma	GSE149614	GSM4505944, GSM4505945, GSM4505947, GSM4505949, GSM4505951, GSM4505953, GSM4505956, GSM4505959, GSM4505961, GSM4505964	32,167
Pancreatic cancer	GSE197177	GSM5910784, GSM5910785, GSM5910787, GSM5910788, GSM5910789, GSM5910790, GSM5910791	37,321
Gastric cancer	GSE183904	GSM5573467, GSM5573468, GSM5573470, GSM5573472, GSM5573473, GSM5573475, GSM5573477, GSM5573478, GSM5573487, GSM5573489, GSM5573491, GSM5573501,	26,193
Colorectal cancer	GSE132465	GSM3868425, GSM3868426, GSM3868427, GSM3868428, GSM3868429, GSM3868430, GSM3868431, GSM3868432, GSM3868433, GSM3868434, GSM3868435	23,171
Total			167,669

## Data Availability

The datasets analyzed in this study can be found in GEO (https://www.ncbi.nlm.nih.gov/geo/ (accessed on 7 November 2025)), Xena (https://xena.ucsc.edu/ (assessed on 7 November 2025)) and GSCA (https://guolab.wchscu.cn/GSCA/#/ (assessed on 11 November 2025)). The datasets used and analyzed in this study are available from the corresponding authors of this study upon reasonable request.

## Supplementary Materials

The following supporting information can be downloaded at: https://www.mdpi.com/article/10.3390/ijms27073208/s1.

## Abbreviations

The following abbreviations are used in this manuscript:

CHOL	Bile Duct Cancer
COAD	Colon Cancer
ESCA	Esophageal cancer
LIHC	Liver cancer
PAAD	Pancreatic cancer
STAD	Stomach Cancer
READ	Rectal Cancer
sc-RNAseq	Single-cell RNA sequencing
CAFs	Cancer-associated fibroblasts
mCAFs	Matrix cancer-associated fibroblasts
apCAFs	Antigen-presenting cancer-associated fibroblasts
ECM	Extracellular matrix
EMT	Epithelial–mesenchymal transition
EndMT	Endothelial–mesenchymal transition
AS	Alternative splicing
GSVA	Gene Set Variation Analysis
GO	Gene Ontology
KEGG	Kyoto Encyclopedia of Genes and Genomes
WGCNA	Weighted Gene Co-expression Network Analysis
LASSO	Least Absolute Shrinkage and Selection Operator
DEGs	Differentially Expressed Genes
PCA	Principal component analysis
UMAP	Uniform Manifold Approximation and Projection
OS	Overall survival
DSS	Disease-Specific Survival
PFI	Progression-free interval
DFI	Disease-Free Interval
IC50	Half-Maximal Inhibitory Concentration
PPI	Protein–Protein Interaction
TME	Tumor microenvironment
