# Supplementary material for: A Conserved Fibroblast-Myeloid Gene Signature in Digestive Cancers: Multi-Omics Integration Identifies DCN, COL10A1, CTHRC1, and TREM2 as Candidate Microenvironmental Markers
Source: Int J Mol Sci. 2026 Apr 1;27(7):3208. doi: 10.3390/ijms27073208 (PMC13072812; doi:10.3390/ijms27073208)
Supplement: Supplementary file 1 [file ijms-27-03208-s001.zip › Supplementary Figures.pdf]

Supplementary Table S1. Differential expression analysis across seven digestive cancers.

Please refer to the separate Excel file named “Supplementary Table S1”.

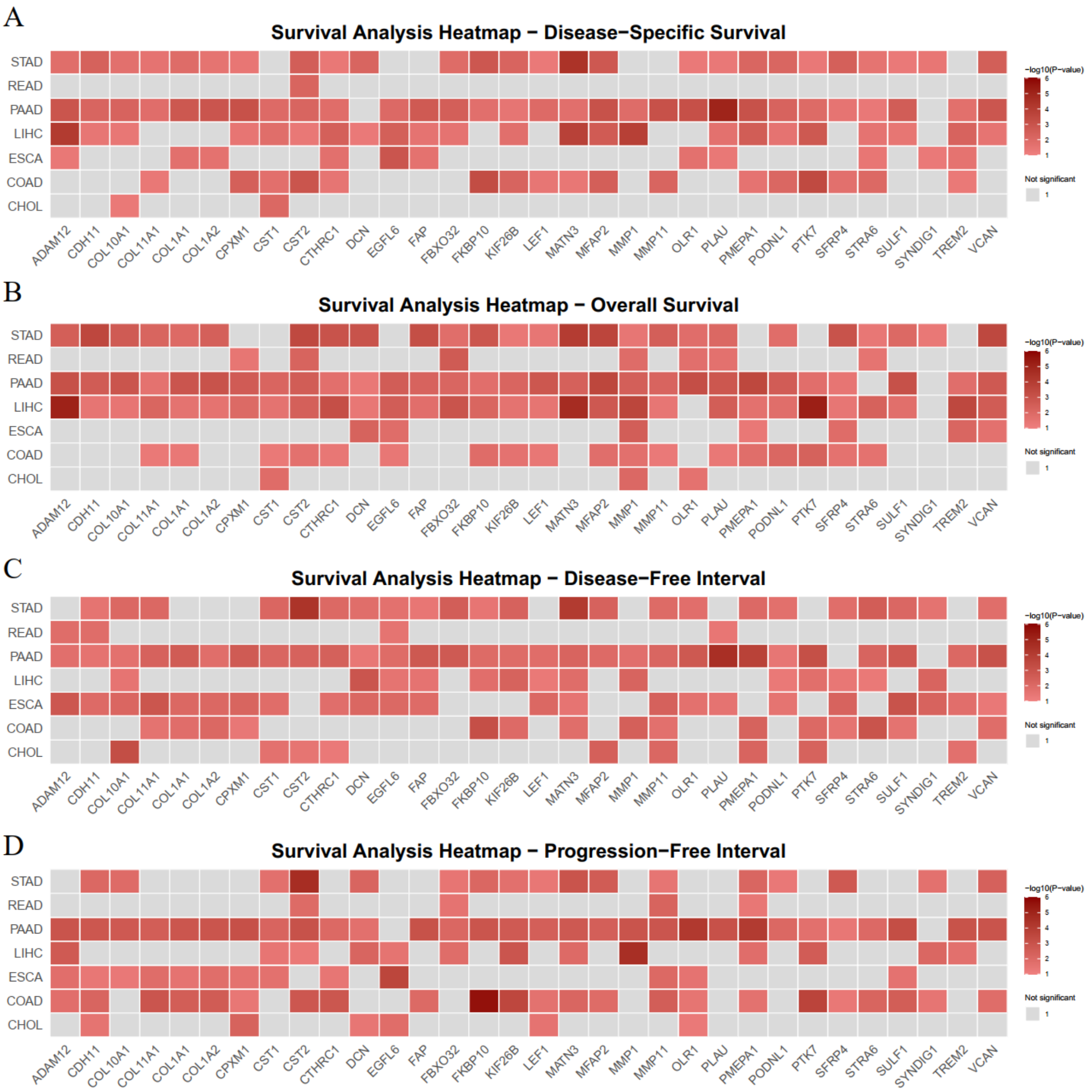

Supplementary Figure S1. Heatmap showing the impact of 32 genes on survival in digestive cancers. Red indicates a significant impact on survival, with darker red representing a more significant P-value. Grey indicates non-significance.(A) Disease-Specific Survival .(B) Overall Survival.(C) Disease-Free Interval.(D) Progression-Free Interval.

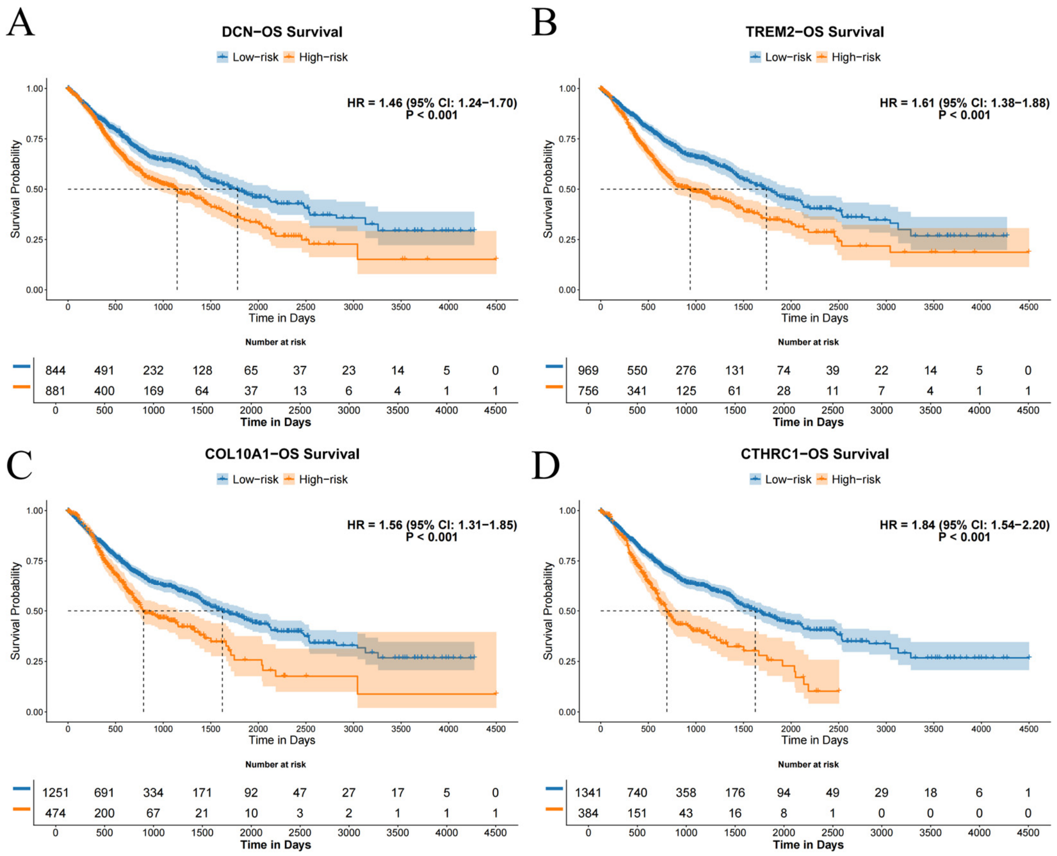

Supplementary Figure S2. Overall survival (OS) curves for DCN, TREM2, COL10A1, and CTHRC1 (stratified by the optimal cutoff value and applied permutation test). **(A)** DCN **(B)** TREM2 **(C)** COL10A1 **(D)** CTHRC1.

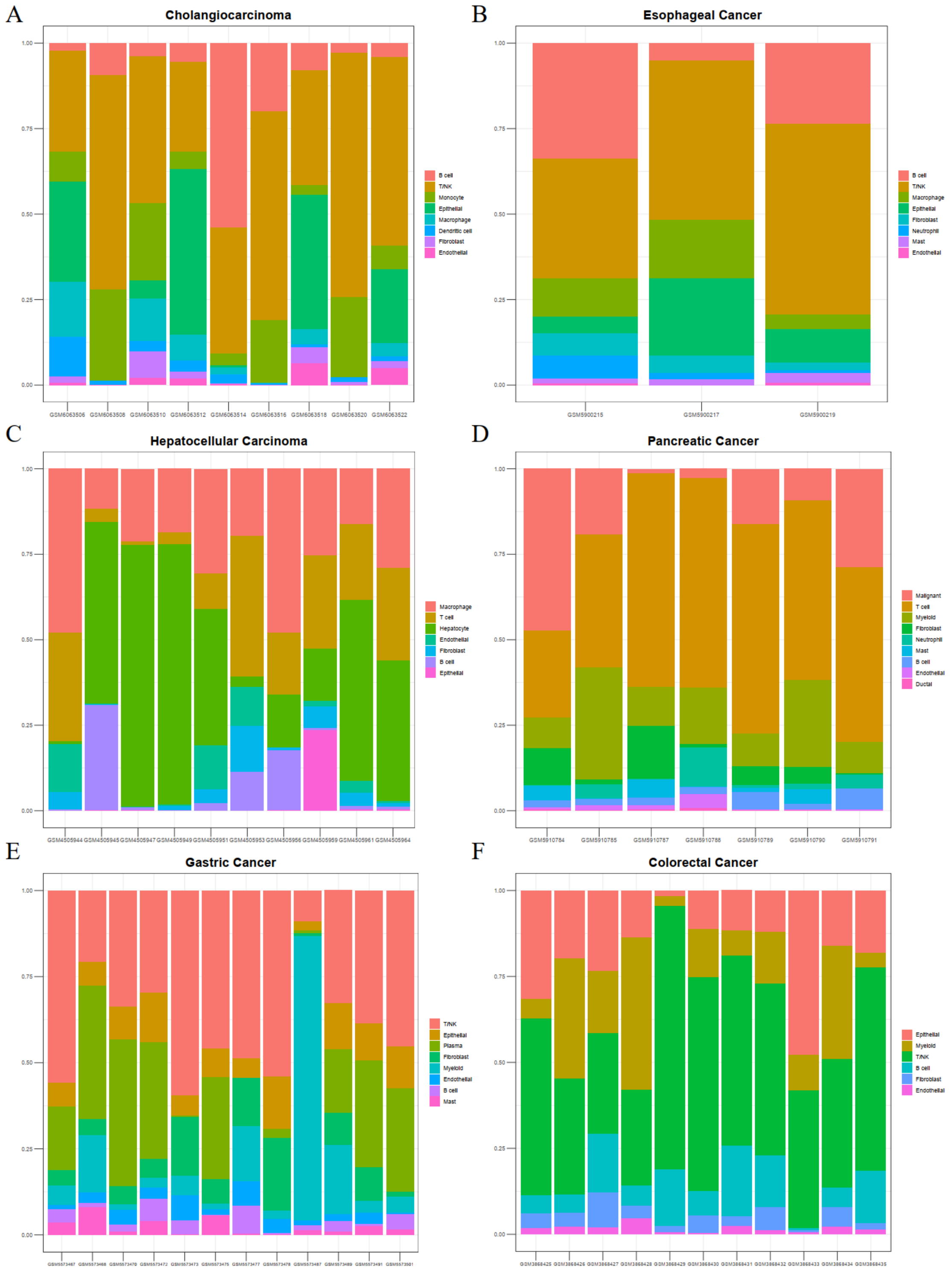

Supplementary Figure S3. Stacked bar plots showing cell type proportions in:(A)Cholangiocarcinoma (B) Esophageal carcinoma (C) Hepatocellular carcinoma (D) Pancreatic cancer (E) Gastric cancer(F) Colorectal cancer

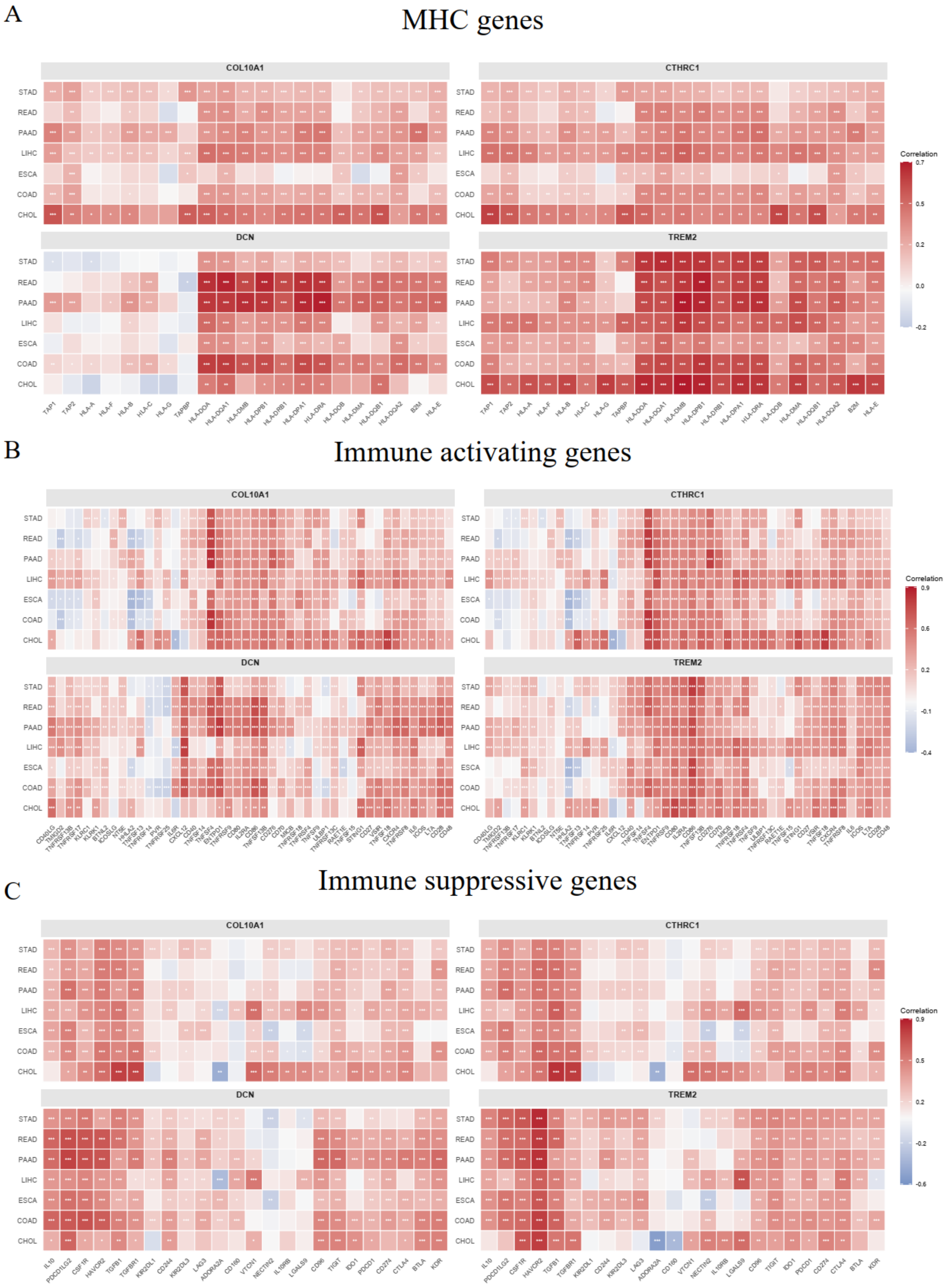

Supplementary Figure S4. Heatmaps showing the correlation of DCN, COL10A1, CTHRC1, and TREM2 with: (A)MHC-related genes(B) Immune activator genes(C) Immune suppressor genes

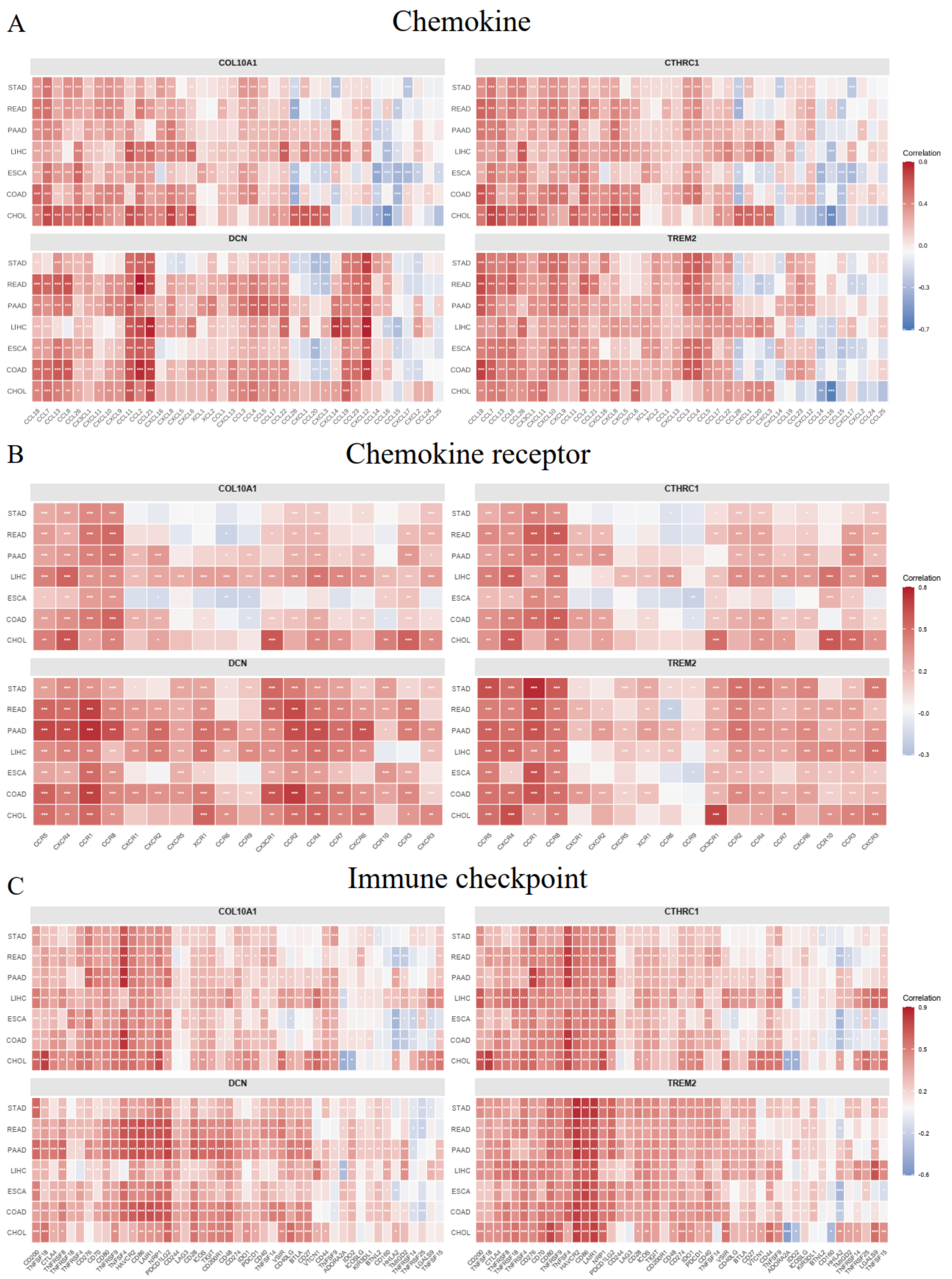

Supplementary Figure S5. Heatmaps showing the correlation of DCN, COL10A1, CTHRC1, and TREM2 with: (A)Chemokines(B) Chemokine receptors(C) Immune checkpoints

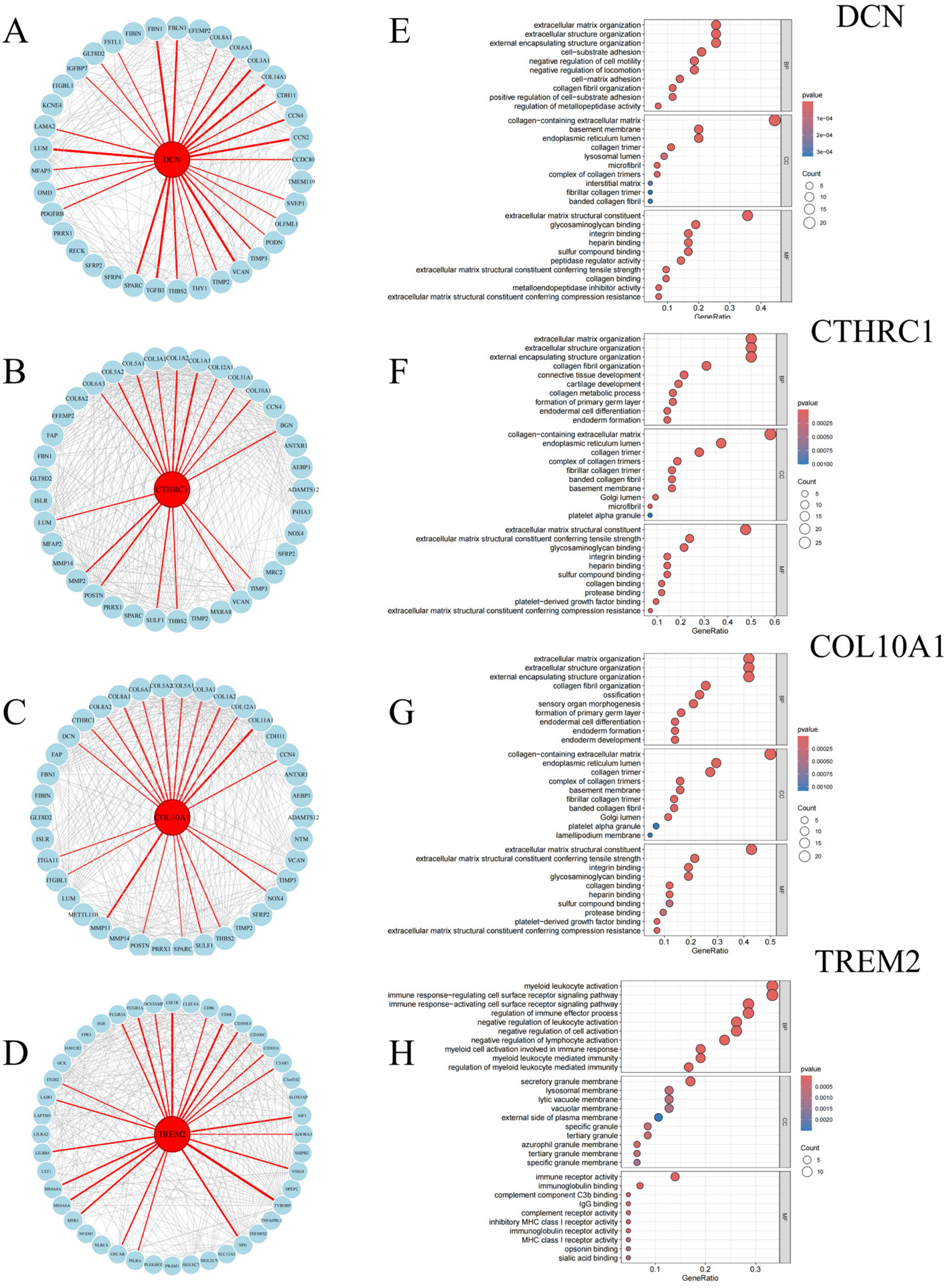

Supplementary Figure S6. Protein-protein interaction and bubble plot of GO enrichment. **(A-D)** Protein-protein interaction (PPI) networks for genes similar to DCN, CTHRC1, COL10A1, and TREM2. **(E-H)** Bubble plots of GO enrichment analysis for genes similar to: DCN, CTHRC1, COL10A1 and TREM2.
