# Supplementary material for: A Conserved Fibroblast-Myeloid Gene Signature in Digestive Cancers: Multi-Omics Integration Identifies DCN, COL10A1, CTHRC1, and TREM2 as Candidate Microenvironmental Markers
Source: Int J Mol Sci. 2026 Apr 1;27(7):3208. doi: 10.3390/ijms27073208 (PMC13072812; doi:10.3390/ijms27073208)
Supplement: Supplementary file 1 [file ijms-27-03208-s001.zip › Supplementary Figures/Supplementary Figure S2.pdf]

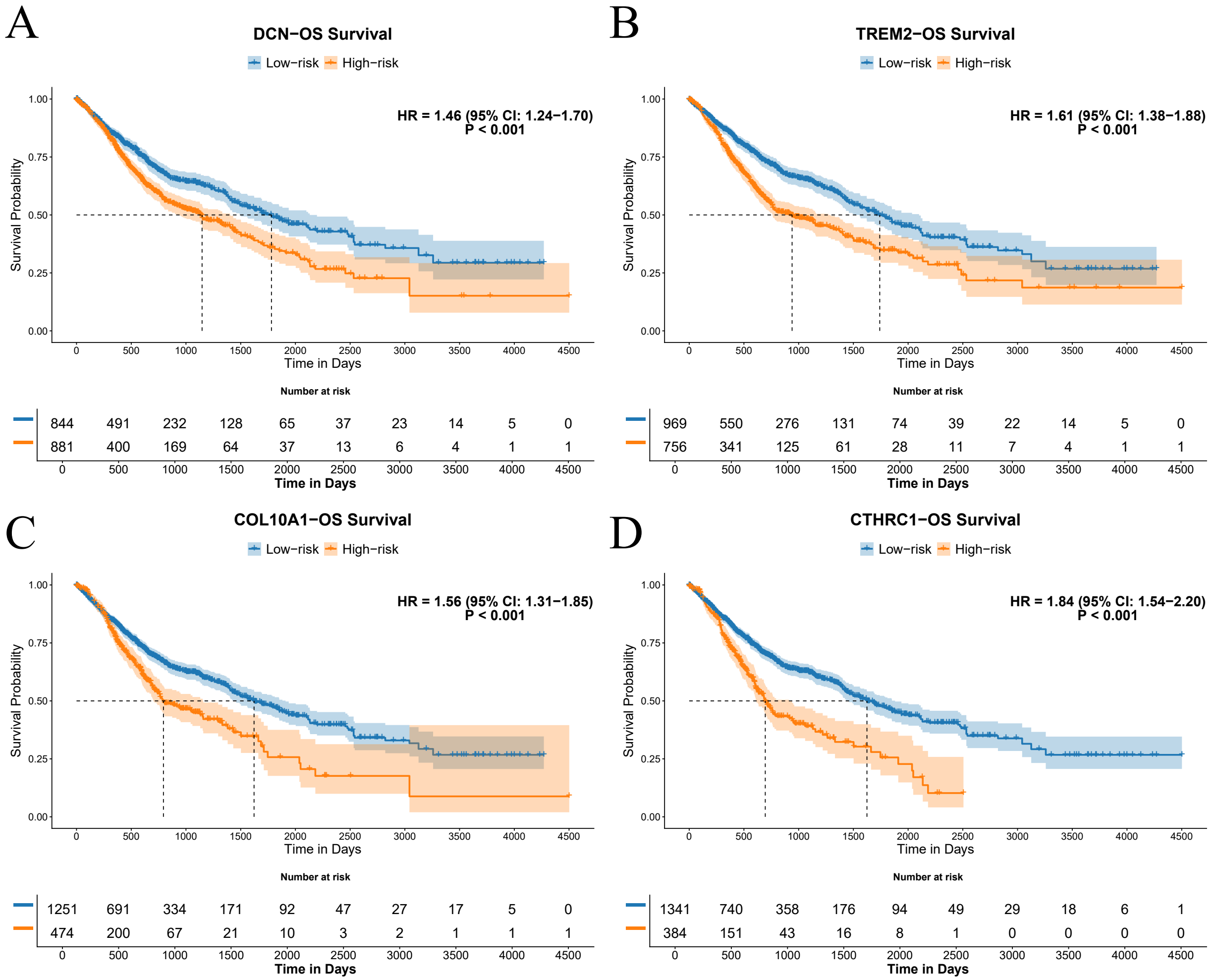
A
DCN−OS Survival
Low−risk High−risk
HR = 1.46 (95% CI: 1.24−1.70)
P < 0.001
Survival Probability
Time in Days
Number at risk
844 491 232 128 65 37 23 14 5 0
881 400 169 64 37 13 6 4 1 1
Time in Days
B
TREM2−OS Survival
Low−risk High−risk
HR = 1.61 (95% CI: 1.38−1.88)
P < 0.001
Survival Probability
Time in Days
Number at risk
969 550 276 131 74 39 22 14 5 0
756 341 125 61 28 11 7 4 1 1
Time in Days
C
COL10A1−OS Survival
Low−risk High−risk
HR = 1.56 (95% CI: 1.31−1.85)
P < 0.001
Survival Probability
Time in Days
Number at risk
1251 691 334 171 92 47 27 17 5 0
474 200 67 21 10 3 2 1 1 1
Time in Days
D
CTHRC1−OS Survival
Low−risk High−risk
HR = 1.84 (95% CI: 1.54−2.20)
P < 0.001
Survival Probability
Time in Days
Number at risk
1341 740 358 176 94 49 29 18 6 1
384 151 43 16 8 1 0 0 0 0
Time in Days
