# Supplementary figures and images for: A Conserved Fibroblast-Myeloid Gene Signature in Digestive Cancers: Multi-Omics Integration Identifies DCN, COL10A1, CTHRC1, and TREM2 as Candidate Microenvironmental Markers
Source: Int J Mol Sci. 2026 Apr 1;27(7):3208. doi: 10.3390/ijms27073208 (PMC13072812; doi:10.3390/ijms27073208)

A

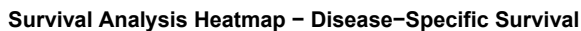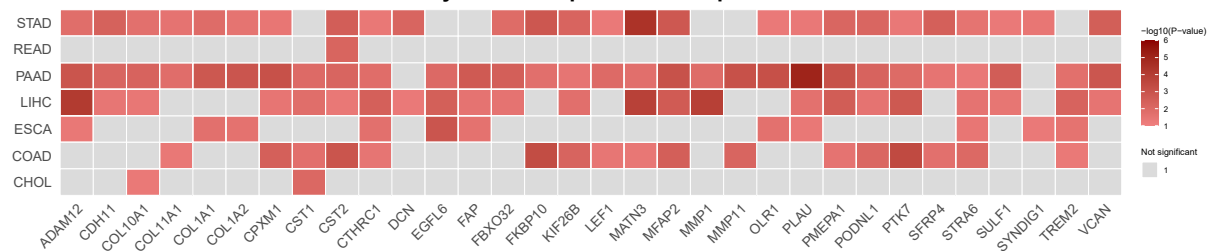

B

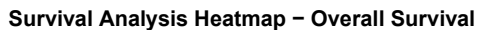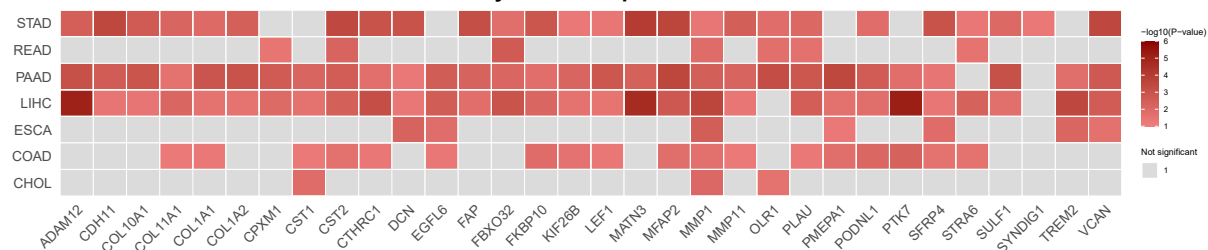

C

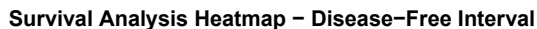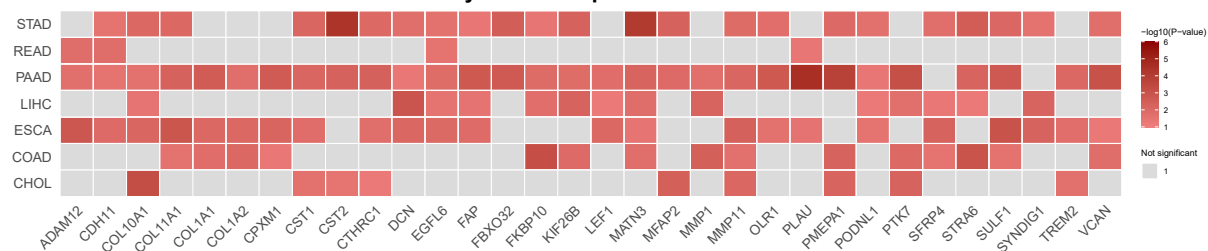

D

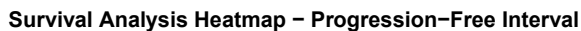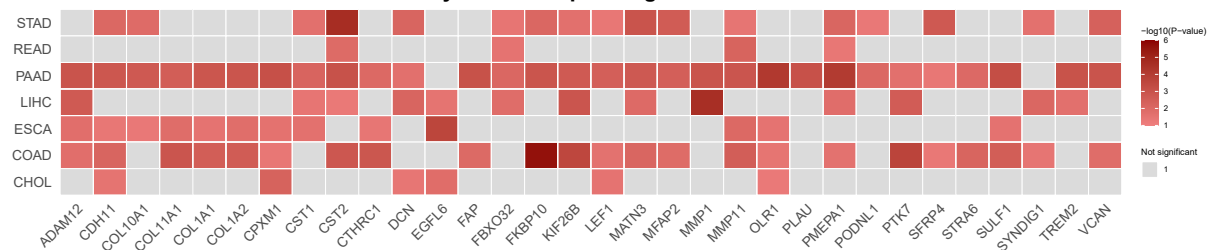

Supplement: Supplementary file 1 [file ijms-27-03208-s001.zip › Supplementary Figures/Supplementary Figure S1.pdf]

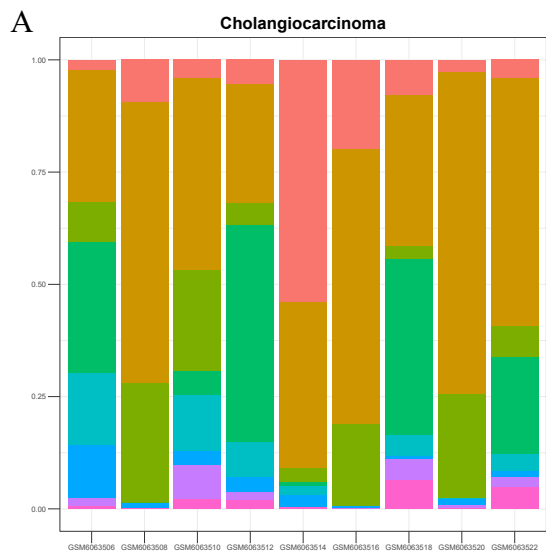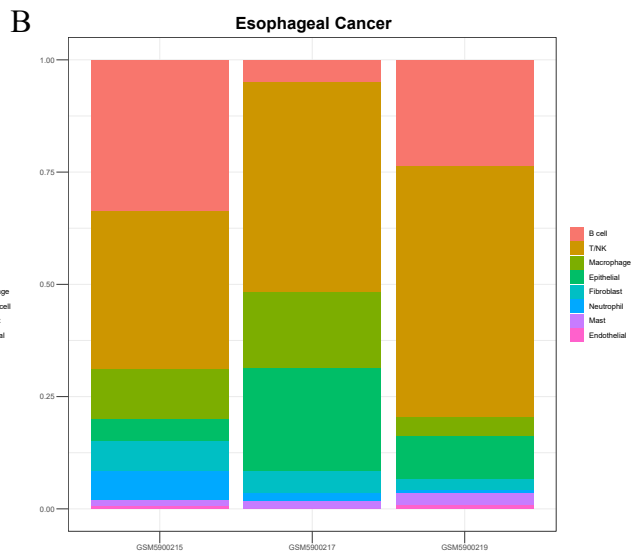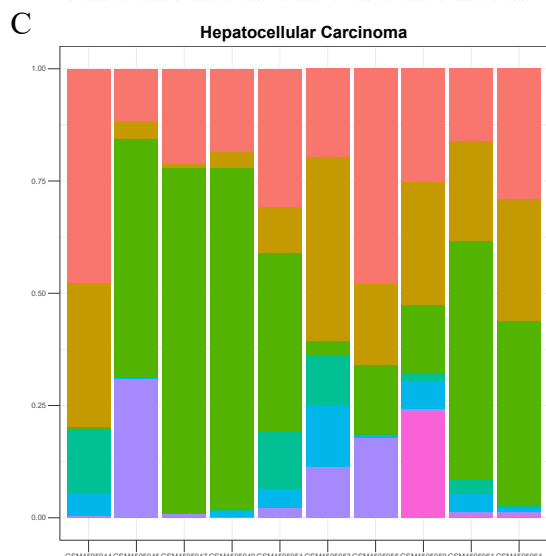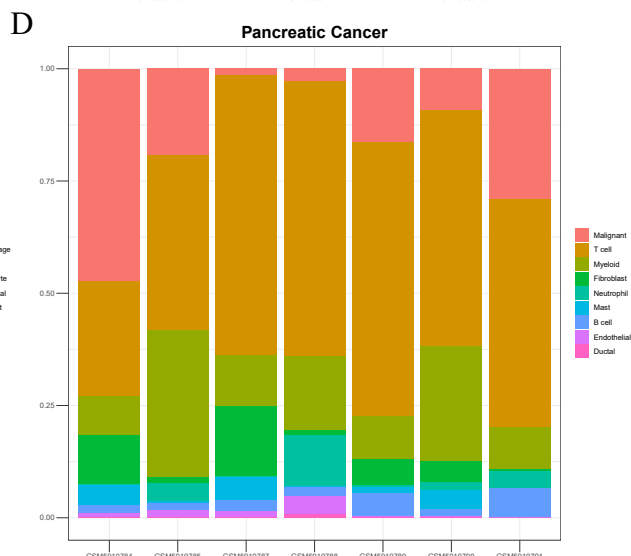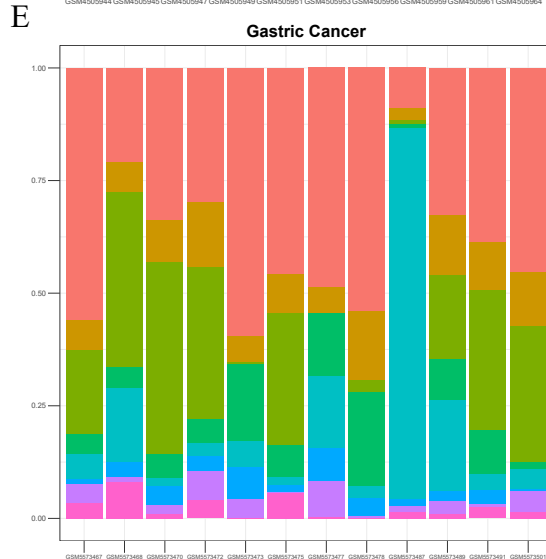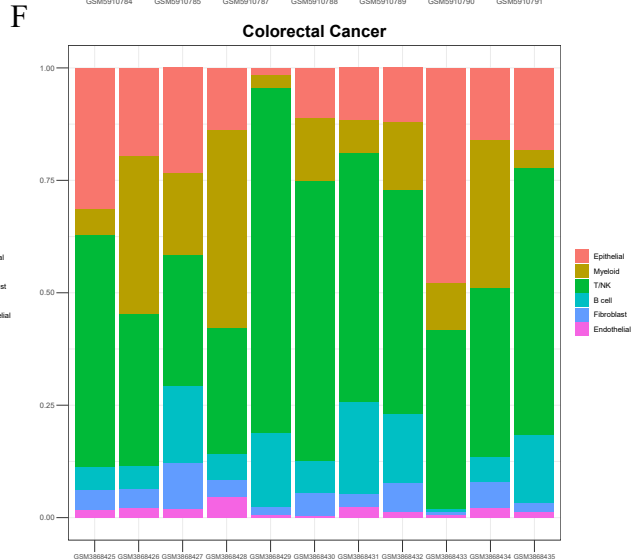

Supplement: Supplementary file 1 [file ijms-27-03208-s001.zip › Supplementary Figures/Supplementary Figure S3.pdf]

MHC genes

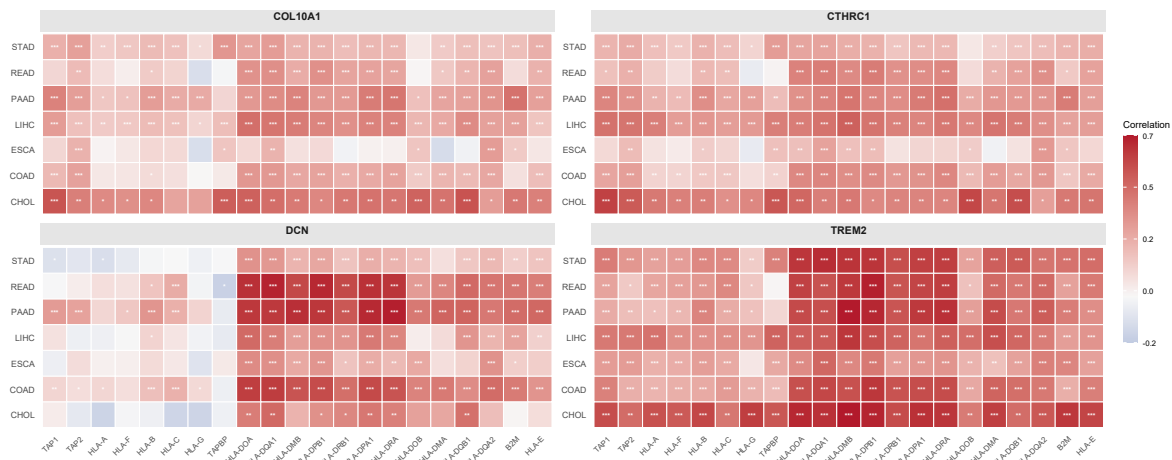

## Immune activating genes

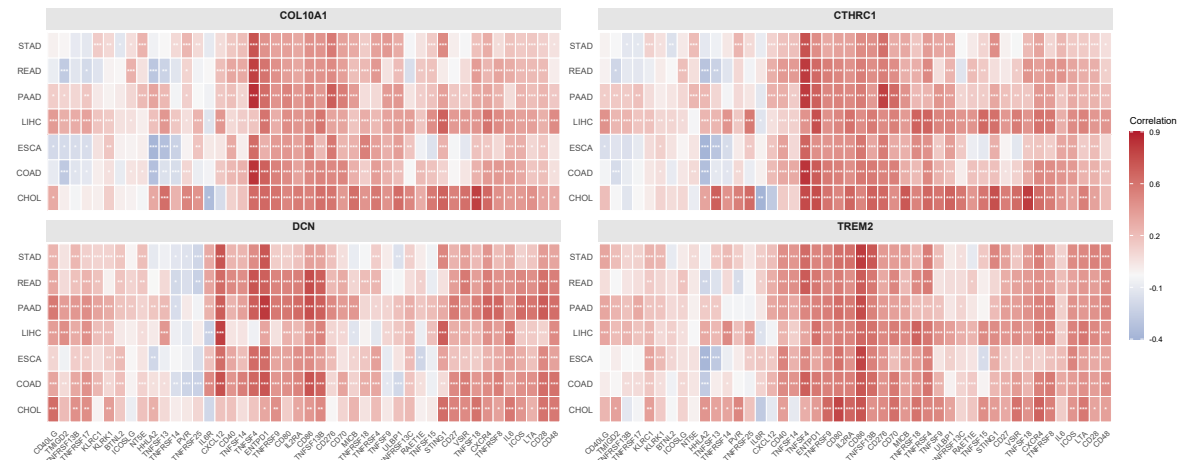

## Immune suppressive genes

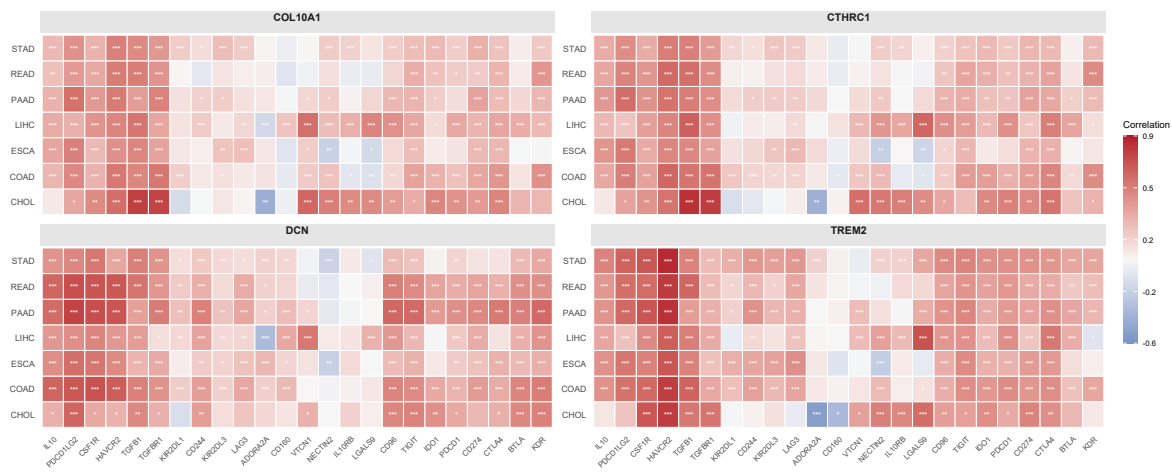

Supplement: Supplementary file 1 [file ijms-27-03208-s001.zip › Supplementary Figures/Supplementary Figure S4.pdf]

## A

# Chemokine

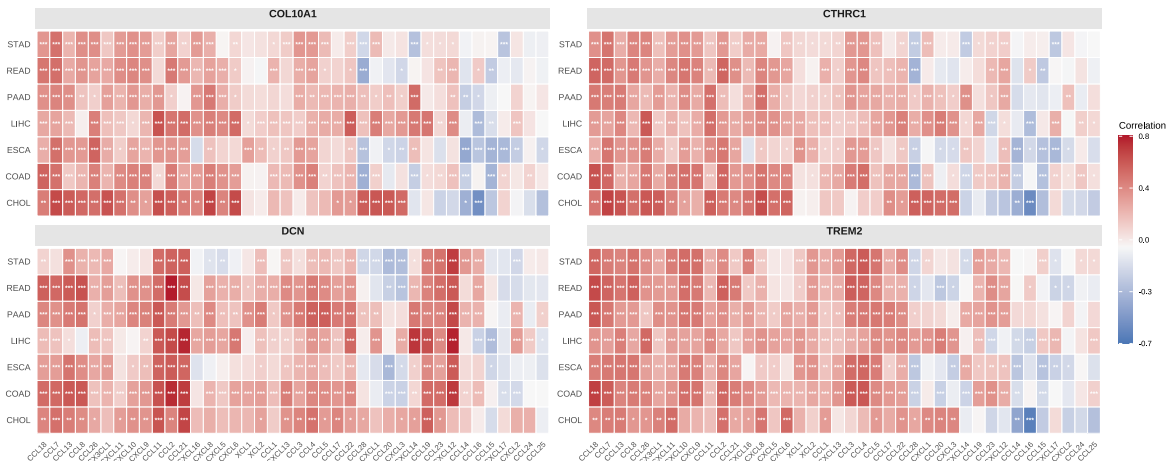

## B

## Chemokine receptor

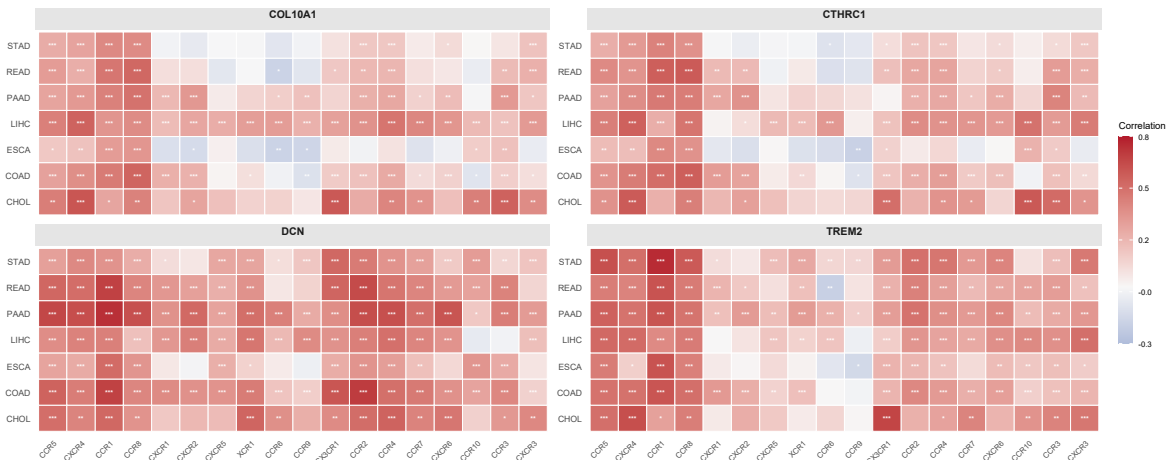

## C

## Immune checkpoint

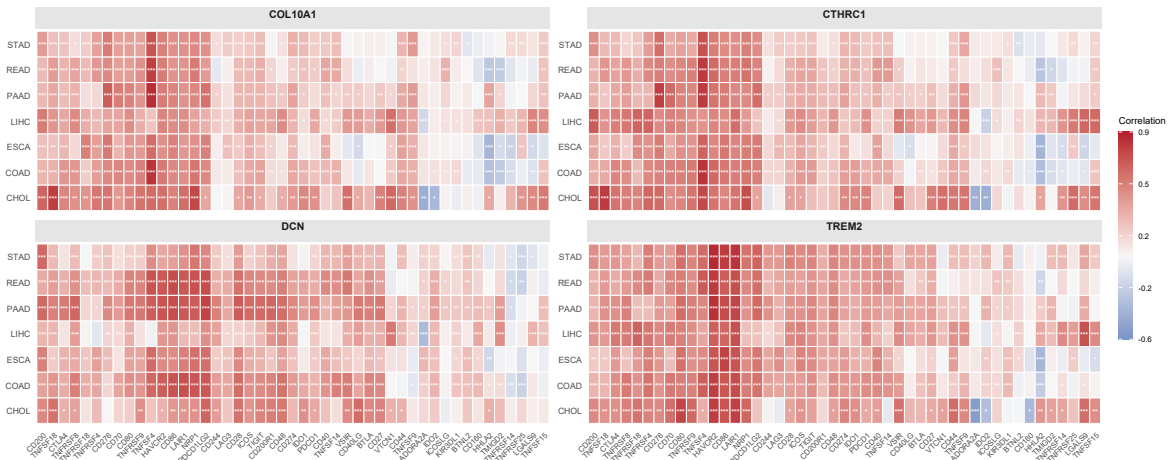

Supplement: Supplementary file 1 [file ijms-27-03208-s001.zip › Supplementary Figures/Supplementary Figure S5.pdf]

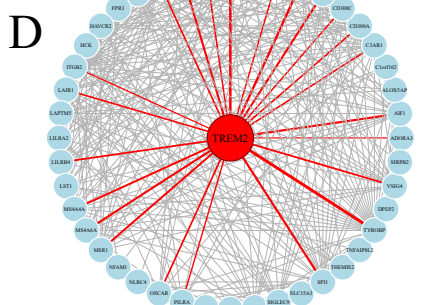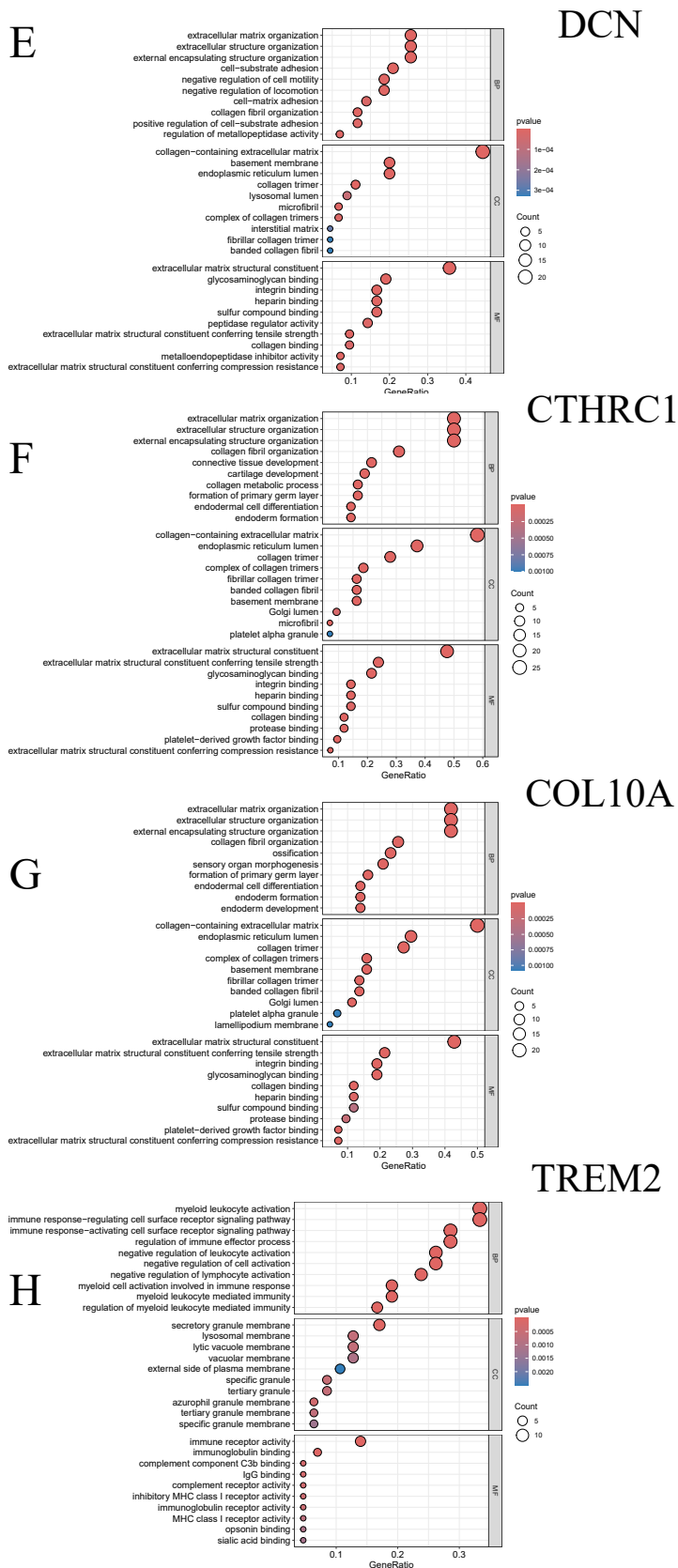

Supplement: Supplementary file 1 [file ijms-27-03208-s001.zip › Supplementary Figures/Supplementary Figure S6.pdf]
